# Generation of an enhancer-driven gene expression viral tool specific to dentate granule cell-types through direct hippocampal injection

**DOI:** 10.3389/fnins.2024.1274174

**Published:** 2024-03-14

**Authors:** Maria Letizia Potenza, Stefan Blankvoort, Miguel M. Carvalho, Joachim S. Grimstvedt, Valentina Di Maria, Kristian Moan, Rajeevkumar Raveendran Nair, Marcus S. Flatset, Qiangwei Zhang, Laurent F. Thomas, Francois P. Pauzin, Rodolfo Da Silva Mazzarini Baldinotti, Giulia Quattrocolo, Clive R. Bramham, Pål Sætrom, Menno P. Witter, Clifford G. Kentros

**Affiliations:** ^1^Kavli Institute for Systems Neuroscience and Centre for Neural Computation, Norwegian University of Science and Technology, NTNU, Trondheim, Norway; ^2^BioCore Bioinformatics Core Facility, Norwegian University of Science and Technology, NTNU, Trondheim, Norway; ^3^Department of Biomedicine, University of Bergen, Bergen, Norway; ^4^Mohn Research Center for the Brain, University of Bergen, Bergen, Norway; ^5^Department of Clinical and Molecular Medicine, Norwegian University of Science and Technology, NTNU, Trondheim, Norway; ^6^Department of Computer Science, Norwegian University of Science and Technology, NTNU, Trondheim, Norway; ^7^Institute of Neuroscience, University of Oregon, Eugene, OR, United States

**Keywords:** rAAVs, dentate gyrus, genetic tool, chromatin immunoprecipitation, stereotaxic injection

## Abstract

Accurate investigations of neural circuitry require specific genetic access to individual circuit elements, i.e., the myriad neuronal cell-types in the brain. However, native promoters cannot achieve this because while most genes are expressed in the brain, few are expressed in a single neuronal cell-type. We recently used enhancers, the subcomponents of the transcriptional apparatus which tell promoters when and where to express, combined with heterologous minimal promoters to increase specificity of transgene expression, an approach we call Enhancer-Driven Gene Expression (EDGE). As we discuss, EDGE is a marked improvement in specificity over native promoters, but still requires careful anatomical analysis to avoid off-target effects. In this study we present a more complete set of genomic markers from the mouse brain and characterize a novel EDGE viral vector capable of specifically driving expression in distinct subtypes of hippocampal neurons, even though it can express in other cell-types elsewhere. The advent of cell-type specific viral tools in wild-type animals provides a powerful strategy for neural circuit investigation and holds promise for studies using animal models for which transgenic tools are not available.

## Introduction

Our ability to understand neural circuits depends on sophisticated molecular and genetic tools that allow targeting of the particular neuronal cell-types. This is truly a daunting task. Few, if any tools exist to target specific cell-types and there is disagreement on how to define cell-types based on morphology, electrophysiology, gene expression and connectivity (Migliore and Shepherd, 2005; Kumamoto and Hanashima, 2014; Sharpee, 2014; Zeng and Sanes, 2017; Cervantes et al., 2019; Hernandez-Perez et al., 2019). The only consensus appears to be that there are many, many more neuronal cell-types than previously thought, possibly over 150 in the retina alone (Siegert et al., 2012; Yamagata et al., 2021) and that obtaining genetic tools specific to them will be crucial to understanding the function of native neural circuits.

The only way to create genetic tools that target specific cell-types is by exploiting the innate regulatory systems for gene expression (Garcia-Marques et al., 2019). Typically, one puts the transgene under the control of a native promoter, which leads to transgene expression wherever the gene is normally expressed (Tsien et al., 1996; Gong et al., 2007; Hernandez-Garcia and Finer, 2014; Danino et al., 2015; Daigle et al., 2018). The problem with this approach is that few genes express in a single neuronal cell-type, so native promoters are simply not specific enough (Feng et al., 2000; Londrigan et al., 2007; Zheng and Baum, 2008; Einarsson et al., 2022). While a gene typically only has one or a few promoters (i.e., sites of actual transcription), distal regulatory elements termed enhancers are several orders of magnitude more prevalent, and since they help determine exactly where and when the promoter is active, they are also likely more specific (Heintzman et al., 2007; Pennacchio et al., 2013; Schoenfelder and Fraser, 2019). This has led us to apply techniques developed by molecular geneticists to study enhancers, allowing us to address the problem of neural diversity, first in transgenic animals (Blankvoort et al., 2018) and then in recombinant adeno-associated viral vectors (rAAVs) (Nair et al., 2020). In both cases we found that enhancer-based expression does indeed achieve much greater specificity than that of native promoters.

**Figure 1:**
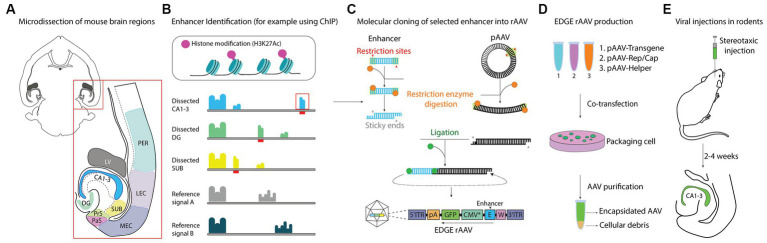
Schematic representation of EDGE workflow. **(A)** Overview of brain regions microdissected and used in ChIP-seq analysis. **(B)** Pipeline for identifying unique enhancer regions in microdissected tissue. H3K27ac provide an epigenetic signature of active enhancers. Genetic data from each region of interest is compared to a reference signal to identify unique signal peaks (indicated by red bars). **(C)** Molecular cloning strategy, showing how single putative enhancers are cloned into a viral backbone containing a heterologous minimal promoter. **(D)** EDGE rAAVs are produced by triple transfection of HEK 293 cells. **(E)** Viruses were administered through local injection (stereotaxic injection) to assess the expression patterns in the brain.

The overall process, which we have termed *Enhancer-Driven-Gene Expression* (EDGE), is schematized in Figure 1. The key is to identify enhancers active only in a brain region or cell-type of interest and combine them with a truncated heterologous promoter. The principles underlying both EDGE and other enhancer-based targeting strategies should be applicable to any part of the brain, given that a suitable enhancer is identified and made into a viral construct. However, at present the tools available to do so are limited to only a few cell-types (Dimidschstein et al., 2017; Blankvoort et al., 2018; Nair et al., 2020; Graybuck et al., 2021; Mich et al., 2021; Lu et al., 2022). To design EDGE based tools for the entire brain is beyond the scope of this paper, but since the main topic of interest for our lab is learning and memory, we have instead focused our efforts on designing tools to investigate regions of the hippocampal formation.

There are a number of methods used to identify enhancers, such as *chromatin immunoprecipitation sequencing* (ChIP-seq) (Park, 2009; Visel et al., 2009; Creyghton et al., 2010; Mundade et al., 2014) and more recently, *assay for transposase-accessible chromatin using sequencing* (ATAC-seq) (Armand et al., 2021; Nakato and Sakata, 2021; Grandi et al., 2022; Preissl et al., 2023). ChIP-seq uses antibodies against various histone modifications associated with different chromatin states to immunoprecipitate regions of the genome. We primarily used H3K27 acetylation, to identify chromatin regions active in regulation of transcription. In comparison, ATACseq uses a transposon to tag regions of open chromatin for further sequencing, and therefore has the downside of not distinguishing between distinct substates of chromatin, meaning that additional screening might be needed. However, it has the very significant upside that it can be done at the single-cell level (scATACseq) (Buenrostro et al., 2015; Fang et al., 2021). This sets it apart from ChIPseq, in which the analyzed bulk tissue does not specify in which particular cell-type the enhancer is active. With scATACseq, one can use accessibility of promoters as a proxy for its level of gene expression. To what extent scATAC-seq can be combined with bulk tissue measures of enhancer-specific histone modifications to identify cell-type specific enhancers is still unclear and beyond the scope of this work.

After enhancer candidates are identified, the next steps are packaging them into rAAVs and screening for the desired expression patterns. We initially did this by making transgenic animals (Blankvoort et al., 2018) and then switched to the far more practical rAAVs screened using local stereotaxic injections into the region of interest (Nair et al., 2020). Although EDGE-rAAVs are indeed more specific than anything else, they are not perfect. We and others (Dimidschstein et al., 2016; Blankvoort et al., 2018; Hrvatin et al., 2019; Juttner et al., 2019; Nair et al., 2020; Graybuck et al., 2021; Mich et al., 2021) have shown how these tools can be used to achieve cell-type specific labeling. However, some level of surgical precision is needed to obtain such specific results. As such, it is crucial to acquire an overview of possible off-target regions these viruses may have, for which one could perform either large volume local injections, or systemic injection strategies. In the present paper, we demonstrate this process with the novel enhancer vHC-20-72, our most recent addition to the toolkit of EDGE-rAAVs, which can be used to target sub-populations of cells in the hippocampal formation. We first screen large areas of the brain to identify targetable regions and then show examples of how this tool can be used more precisely, both in adult and developing mice, and even rats.

## Materials and methods

### Chromatin immunoprecipitation

Putative enhancers were identified using Chromatin Immunoprecipitation followed by high-throughput sequencing (ChIP-seq). ChIP-seq was performed on micro-dissected brain tissues extracted and collected as previously described in our laboratory (Blankvoort et al., 2018). Brain tissue was then cross-linked in 1% formaldehyde for 15 min at room temperature, followed by quenching with glycine (150 mM in Phosphate-buffered saline, PBS) for 10 min. The tissue was then washed by centrifugation at 4°C and the supernatant was removed. Another wash in PBS was performed by centrifugation at 4°C and the supernatant was removed. The tissue was flash-frozen in dry ice and 70% ethanol. Subsequently, the samples were homogenized to isolate nuclei and chromatin was extracted by lysis and sheared by sonication (30 min, 10 s pulses with 10 s rest, 60-70 W total intensity, approximately 15-20 W per Eppendorf tube). For each ChIP-seq experiment, 1-10 mg of final soluble chromatin was combined with coated magnetic beads (Protein G Dynabeads, Invitrogen, cat# 10004D) prebound with 5 μg of antibodies to H3K4me2 (Abcam ab7766) or H3K27ac (Diagenode, C15410196). Five washes with 1 mL of wash buffer and one wash with Tris-EDTA (TE) buffer were executed to immunoprecipitate the chromatin (Cotney and Noonan, 2015; de Jonge et al., 2020). ChIP DNA was separated from protein by reverse cross-linking, with an overnight incubation at 65°C in a thermoshaker (800 rpm), and then purified. Libraries were prepared for sequencing using Rubicon genomics Thruplex DNA-seq kit (R400427, single index). Sequencing was performed on the Illumina HiSeq 2000 platform at the New York Genome Center for Genome Analysis (NYGC) and the Illumina NextSeq 500 platform at the genomics core facility of NTNU. In addition to the 19 tissue samples collected here, we used data from Blankvoort et al. (2018, GSE112897) and Shen et al. (2012)(GSE29184). Further analysis was done as described in Blankvoort et al.(2018). For full lists of the genomic locations of tissue specific, unique putative enhancers, see (Supplementary Table S1).

### Molecular cloning of EDGE rAAV constructs

Due to their small size, enhancers can be cloned into recombinant adeno-associated viruses (rAAVs) and used together with a minimal promoter of 134 base pairs (bp) to achieve highly specific viral expression. The rAAV is then injected into rodent brain for anatomical characterization of cell-selectivity. Our molecular cloning strategy involved PCR from mouse genomic DNA, followed by sticky-end ligation-based cloning. The EDGE rAAV construct was generated using a backbone plasmid pAAV-PolyA-eGFP as previously described in our laboratory (Nair et al., 2020). We used two primers synthesized by Invitrogen (Thermo Fisher Scientific): a forward (5’-GTGTACGAATTCTAATAGAAACTGTTTGCTATGT-3′) primer containing the restriction enzyme EcoRI (NEB™ R0101S) and a reverse (5′- GTGTACGTCGACTTGTATTGTAACATAGACTCTCACCT-3′) primer containing the restriction enzyme SalI (NEB™ R0138S) used for the amplification of the enhancer sequence of interest. Primers were dissolved in RNase free water at a stock concentration of 100 μM. For the PCR reaction, the following reagents were mixed and filled up with RNase free water to a total volume of 25 μL: 1 μL of genomic DNA (1 ng/μl final concentration), 1.25 μL of each primer (0.5 μM final concentration for each primer), 12.5 μL of Q5 Fidelity Master Mix (NEB, #M0492S). PCR was performed using a thermocycler (BIO-RAD T100™) with an initial step of 30 s at 98°C, then 35 cycles of 10 s at 98°C, 30 s at 57°C, 30 s at 72°C and 2 min at 72°C. DNA samples were loaded on a 1% agarose gel and run at 90 V for 1 h and half in Tris-acetate-EDTA (TAE) buffer. The separated DNA fragments were visualized at 365 nm using an UV transilluminator (Syngeng™) and rapidly cut to minimize the UV exposure. DNA extraction from the gel was performed using QIAquick gel extraction kit (Qiagen™ Cat. No. 28706) and the DNA concentration was determined using a Nanodrop One (ThermoScientific). DNA inserts and the viral backbone were separately digested with the same restriction enzymes and the vector plasmid was dephosphorylated at 5′ and 3′ ends using an alkaline phosphatase enzyme (Rapid DNA dephos and ligation kit, Roche Diagnostics, Ref. 04898125001) to reduce the risk of self-ligation (Hoseini and Sauer, 2015). After the ligation reactions, the DNA was used to transform chemically competent *E. coli* cells (One shot Stbl3, Invitrogen by ThermoFisher Scientific) using the heat-shock procedures, which led to the generation of several colonies on agar plates containing ampicillin (100 μg/mL). A fast plasmid mini-preparation kit (QIAprep spin Miniprep Kit, QIAGEN) was used to extract the plasmid from the bacterial suspension. Plasmid DNA samples were screened by digestion with restriction enzymes and clones containing the enhancer sequence of interest were confirmed by DNA sequencing. For subsequent EDGE rAAVs preparations, large-scale plasmid purification was performed using a maxi-preparation kit (QIAGEN, #12663).

### Production and titration of EDGE rAAVs

EDGE rAAVs was packaged in the serotype rAAV 2/1 having a mosaic of capsid 1 and 2 (Nair et al., 2020) used for stereotaxic injections. Briefly, Human Embryonic Kidney cells (HEK 293 T cells, ATCC, CRL-3216) were thawed, split every other day for one or two weeks, cultivated in DMEM (Gibco™ 61965059) containing 10% volume/volume (v/v) fetal bovine serum (FBS, Biological Industries™; cat no. 04–007-1A) and 1% (v/v) penicillin/streptomycin (P/S, Gibco™ Ref: 15140_122, Life Technologies) and grown in log phase in a humidified incubator at 37°C with 5% CO_2._

To package the construct into rAAV 2/1, 7.3 × 10^6^ HEK 293 T cells were seeded the day before transfection into 150 mm cell culture dishes in DMEM medium containing 10% (v/v) FBS and 1% (v/v) P/S. Lipofectamine (Invitrogen™ #11668019) mediated co-transfection in OPTIMEM (Gibco™ 11058–021) with pAAV-containing the transgene (22.5 μg), pHelper (22.5 μg), pRC (11.3 μg) and pXRI (11.3 μg) capsid plasmids, was performed next day, with cells at about 80% confluence. After 24 h, the medium was replaced with fresh DMEM containing 1% (v/v) P/S and 48–72 h later, the EDGE rAAV was extracted using Heparin column affinity purification method (Nair et al., 2020). Infected cells were scraped off and centrifuged at 200 x g for 10 min, and the pellet was treated with a buffer containing 150 mM NaCl, 20 mM Tris pH 8.0 and 10% sodium deoxycolate. Subsequently, the pellet was added Benzonase nuclease HC (Merck Millipore, 71206–3) to a final concentration of 50 units per ml and the lysate was incubated for 1 h at 37°C. Cellular debris were then removed by centrifuging the lysate at 3000 x g for 15 min and using a peristaltic pump the supernatant was subjected to HiTrap® Heparin High Performance (GE, 17–0406-01) affinity column chromatography. Finally, the elute from the Heparin column was concentrated using Amicon Ultra-4 centrifugal filters (Merck Millipore, UFC810024).

The viral titer of the EDGE rAAV was determined by quantitative PCR (qPCR) using Power SYBR™ Green PCR Master Mix and a StepOne machine (Applied Biosystems, USA). At first, a standard curve was generated, preparing 5 serial dilutions of linearized plasmid (from 10^9^ to 10^5^). Subsequently, purified rAAV vectors and standard curve DNA samples, were quantified using ITR primers (CGGCCTCAGTGAGCGA ITR_F; GGAACCCCTAGTGATGGAGTT ITR_R). The qPCR reaction was done by an initial step of 30 s at 98°C, then 39 cycles of 10 s at 98°C, 30 s at 60°C, 15 s at 95°C and 1 min at 60°C; followed by a final 15 s step at 95°C. The resulting viruses had a titer of approximately 10^12^–10^13^ viral genomic particles/ml. Detailed information about the viral titer of each EDGE-rAAV can be found in the supplementary material (Supplementary Table S1).

### Animals and husbandry

All animals were group housed in environmentally enriched cages with a 12:12 h reversed light/dark cycle and had *ad libitum* access to food and water. Both male and female wild-type (*N* = 12) C57BL6/J^1^ and C57BL/6JBomTac^2^ strains were used for enhancer screening experiments and subsequent characterization of the selected enhancer. Additionally, to characterize the expression patterns of the EDGE rAAV virus in postnatal development, we used a transgenic cross between the Rbp4-cre line^3^ and a tdTomato reporter line,^4^ to label neurons in dentate gyrus. To do so, 2 litters (*N* = 13 pups in total) were injected at postnatal day zero (P0). Tail tissue was used to genotype *Rbp4*-Cre positive (*Rbp4*-Cre^+^) and tdTomato positive (*tdTom+*) and negative littermates were used as control animals. We also used male Sprague Dawley rats^5^ between 3 and 6 months of age (*N* = 8), for the histological assessment of transgene expression of the EDGE rAAV virus in the dentate gyrus of another rodent species.

All experiments using mice were conducted at the Kavli Institute for Systems Neuroscience-Centre for Neural Computation at the Norwegian University of Science and Technology (NTNU), while experiments using rats were performed at University of Bergen, Department of Biomedicine. The Norwegian Food Safety Authority (FOTS) approved all experimental procedures, which have been performed in accordance with the Norwegian Animal Welfare Act and the European Convention for the Protection of Vertebrate Animals used for Experimental and Other Scientific Purposes. Comprehensive details regarding the dataset of injected mice can be found in the supplementary material (Supplementary Table S1).

### Surgical procedure and viral injection

Mice that underwent stereotaxic surgery, were first anesthetized with 5% isoflurane in an induction chamber (airflow: 1 L/min). They were then head-fixed to a stereotaxic frame and placed on a heating pad at 37°C throughout the whole surgery. Analgesia was provided by subcutaneous injections of Temgesic (0.1 mg/kg) and Metacam (1 mg/kg). Additionally, to protect the animal from dehydration, saline injections were administered during the surgery and eye ointment was applied to the eyes of the animal. Before making a rostrocaudal incision of the cranium, mice were injected with a local anesthetic (Marcaine® 1 mg/kg) and the surgical area was disinfected with ethanol (70%) and iodine. After leveling the skull between bregma and lambda; a craniotomy was made around the coordinates for the injection and precise measurements were performed with the 0.5-μL Hamilton syringe (Hamilton, Unites States) or the glass capillary (World precision Instruments 1.14MM 3.5, #504949) used for the viral injection. The coordinates of the injection sites are as follows: dDG (AP: −2.1 mm, ML: +/− 1.5 mm, DV: −2.2 mm); dCA1 (AP: -2 mm, ML: +/− 1.5 mm, DV: −1.45 mm); dSUB (AP: −3.4 mm, ML: +/− 1.8 mm, DV:-1.4 mm); mPFC (AP: 1.9; ML: 0.5; DV: 1.9). For post-operative care, analgesia was administered 7–12 h after surgery and the weight of the animals was monitored for the following 3 days.

A total of 13 pups of the Rbp4-Cre mouse line were bilaterally injected with EDGE rAAV 2/1 vHC-20-72-eGFP at postnatal day zero (P0). Prior to stereotaxic surgery, pups were anesthetized with between 3 and 5% isoflurane and their head-fixed in a stereotaxic frame (Kopf) equipped with a custom-made adaptor. An ordinary laboratory tape with a diamond cut was applied on the head to stretch the skin. Injection coordinates were calculated from lambda (AP: +0.8; ML: ±1.2; DV: −1.22) in each mouse. The injections were then performed with a virus-filled glass pipette (Drummond) attached to Nanoject III injector (Drummond Scientific Company). Specifically, 25 nL of EDGE rAAV 2/1 vHC-20-72-eGFP was injected in the left hemisphere (rate 1 nL/s) and 50 nL of EDGE rAAV 2/1 vHC-20-72-eGFP (10^12^ viral genomic particles/ml) was injected in the right hemisphere (rate 2 nL/s).

Sprague Dawley rats (*N* = 8) were anesthetized with Domitor (0.3 mg/kg) and Fentanyl (0.3 mg/kg) by intraperitoneal injection, prior to stereotaxic injections. When animals no longer showed any responsiveness (i.e., in the absence of a reaction to toe pinch), they were transported to the stereotaxic frame (David Kopfs Instrument, United States) and temperature was maintained at 37°C. Additionally, to protect the animal from dehydration, saline injections were administered during the surgery and eye ointment was applied to the eyes of the animal. After leveling the skull between bregma and lambda a craniotomy was made around the coordinates for the injection and precise measurements were performed with a 10-μL Hamilton syringe (Hamilton, United States) used for the viral injection. The coordinates used to hit the DG hilar region were the following: AP -3.9 mm; ML +/−2.2 mm; DV -3.3 mm. We injected 1 μL of EDGE rAAV 2/1 vHC-20-72-eGFP (10^12^ viral genomic particles/ml) or rAAV hSyn-Arc-N-lobe at a rate of 10 μL/h. The needle was left in place for 5 min post-injection before being pulled out of the brain slowly and gradually (50 μm every 20 s for 3 min, then 100 μm every 10 s until out of the brain) to keep the rAAV solution at the site of injection. The wound was sutured using Tissue Adhesive (3 M Vetbond™, USA). Then, the animal was transported to its home-cage and was placed on a heating blanket until awake. The animal was given easily digestible food during recovery. After waking up from anesthesia, rats were returned into the housing room and followed-up for wound healing and any signs of pain or discomfort for at least 3 days. During the next 3 days following surgery, Buprenorphine (analgesic) was administered (0.05 mg/kg, subcutaneously) and documented on the cage card.

### Histology and tissue acquisition

After an appropriate survival time for each experiment (2 weeks after stereotaxic injections, postnatal day 10 and 30 after viral injections in P0 pups) the animals were euthanized to collect tissues used for subsequent histological analysis. Briefly, after anesthesia by inhalation of isoflurane and an intraperitoneal injection of pentobarbital (Norges Apotekerforening, 100 mg/mL, 1 mL, or Pentoject, 100 mg/mL, 1 mL), each animal was transcardially perfused with saline and with freshly made 4% paraformaldehyde (PFA). The brains were then removed from the skull and post-fixed for 24 h in 4% PFA solution, at 4°C. Brains were subsequently transferred into a cryoprotective solution containing 20% glycerol and 2% dimethyl sulfoxide (DMSO) diluted in 0.125 M phosphate buffer and stored at 4°C. In some experiments, brains were instead transferred in 15% sucrose solution overnight (4°C) and then moved to a 30% sucrose solution for an additional overnight (4°C). A freezing microtome (Thermo Scientific™ HM 430, USA) was used to cut the brains into 40-μm or 50-μm-thick coronal sections, which were collected in four equally spaced series for tissue processing and stored at −20°C in cryoprotective solution. Immunohistochemistry was done using standard procedures for free floating sections, starting with three initial 10 min washes in 0.125 M phosphate buffer (PB), and subsequent permeabilized by two 10 min wash in 0.125 M PB with 0.5% TritonX (PBT) (Sigma, Cat#T9284); at room temperature (RT). To avoid unspecific antibodies (AB) binding, blocking was done with 5% normal goat serum at RT for 90 min. The brain sections were then incubated for 48 h at 4°C with primary antibody (AB). The following primary ABs were used: Rabbit-anti-Calbindin D-28 K (1:800, Swant, #CB8), Rabbit-anti-Prox1 (1:500, Abcam, #101851), Chicken-anti-eGFP (1:800, Abcam, #13970), Guinea pig-anti-NeuN (1:1000, Millipore, #ABN90P). Secondary incubation with Alexa Fluor AB was done for 2 h at room temperature. The following secondary ABs were used: Goat anti-Rabbit Alexa Fluor 546 (1:400, Life Technologies, #A11010), Goat anti-Chicken Alexa Fluor 488 (1:400, Life Technologies, #A11039), Goat anti-Guinea pig Alexa Fluor 647 (1:400, Life Technologies, #A21450). An alternative protocol was used on some of the brain sections within the study on early postnatal development. Free floating sections were washed two times for 10 min in phosphate-buffered saline (PBS). Blocking was done with 5% normal donkey serum (Sigma #D9663) for 2 h at RT, followed by 3 days of primary incubation at 4°C with Chicken-anti-eGFP (1:1000, Abcam, #13970), Guinea pig-anti-NeuN (1:1000, Millipore, #ABN90P), Mouse-anti-GFAP (1:1000, Millipore, #MAB360), Goati-anti-Iba1 (1:1000, Abcam, #5076), Rat-anti-RFP (1:1000, Chromotek #5f8). Secondary antibody incubation was done overnight at RT using Donkey anti-Chicken Alexa Fluor 488 (1:500, Sigma #sab 3,700,213), Donkey anti-Guinea pig Alexa Fluor 405 (1,250, Jackson Immuno Research #706–475-148), Donkey anti-Mouse 647 (1,500, Invitrogen #A31571), Donkey anti-Goat 633 (1,500, Invitrogen #A21082), Donkey anti-Rat 594 (1,500, Jackson Immuno Research #712–065-153). Sections were mounted on microscope slides (Menzel-Gläser SuperFrost®Plus) and after a drying step, they were cleared for 10 min in Toluene and cover slipped in a mixture of Toluene and Entellan (Merck KGaA).

### Image acquisition and analysis

Single images and z-stacks of the region of interest (ROIs) were acquired at 20X/0.8 NA M27 objective with a confocal microscope (Zeiss LSM 880755 AxioImager Z2) from the mounted brain sections. Images were processed with ZEN Black 2.1 SP2 and ZEN Blue 2.3 Lite, to increase the signal quality and then imported to Adobe Illustrator CS6 (Adobe Systems Incorporated) to design final figures. Adjustments made to improve signal was always applied to the entire image. Confocal images containing the ROIs were analyzed in Neurolucida (Micro Bright Field Bioscience) and cell counting analysis was done with Neurolucida Explorer 776 (Micro Bright Field Bioscience). Histological quantifications were done in Microsoft Excel.

### Data availability statement

The FASTQ files for the selected brain regions as well as a countable containing relative peak strength are available on the GEO online repository with the following ID: GSE240042 (https://www.ncbi.nlm.nih.gov/geo).

## Results

### Experimental pipeline for constructing region specific, enhancer driven viral tools

**Figure 2:**
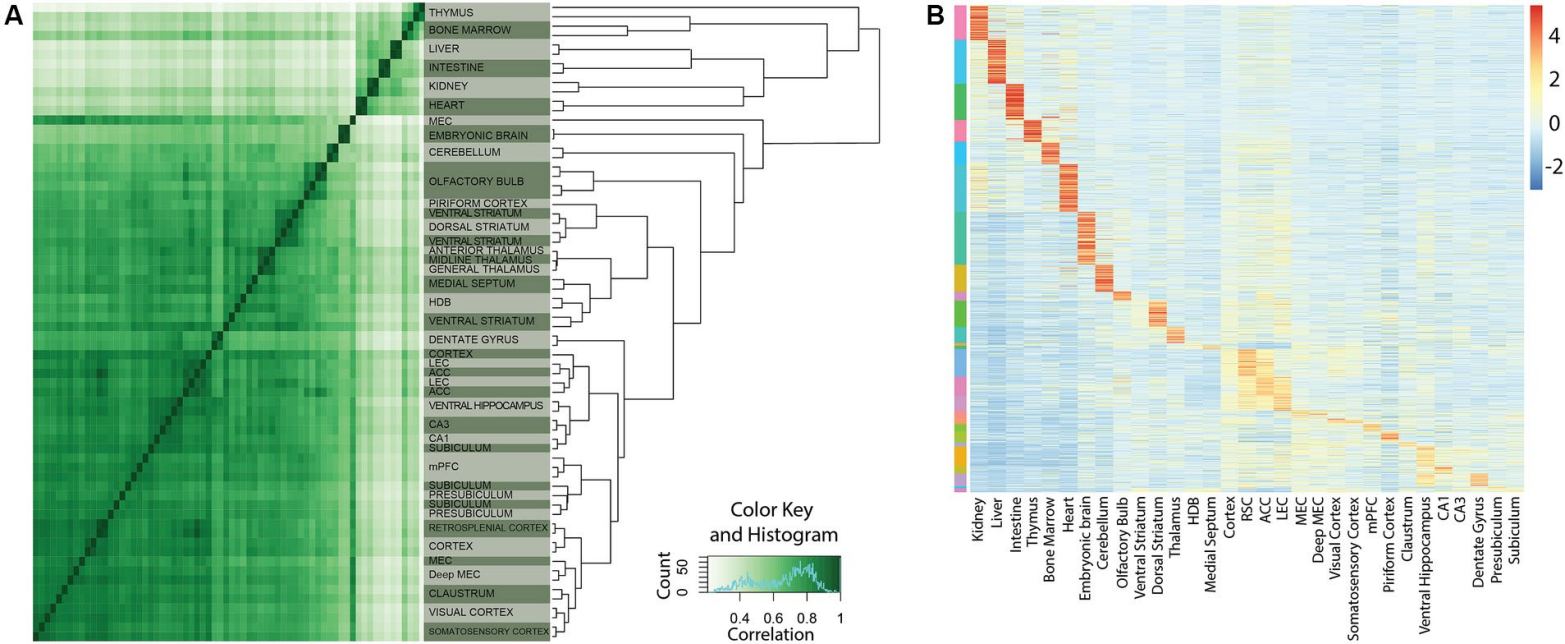
Unique cortical enhancers identified by ChIP-seq. **(A)** Correlation of genome-wide H3K27Ac signals of all tissue samples used in this study. The heatmap on the left shows pairwise correlations of all tissue samples. The dendrogram on the right represents hierarchical clustering of the tissue samples based on their H3K27Ac signal. Note that the majority of samples cluster together between replicates. **(B)** Relative activity of all enhancers identified in this study. The heatmap of 150,289 rows (representing a unified list of putative enhancer regions) by 31 tissue samples are based on Z-scores representing relative H3K27Ac signal strength. The scalebar indicates Z-score. The color bar on the left indicates assignment of putative enhancers to the different tissues. Assignment is based on K-means clustering.

**Figure 3:**
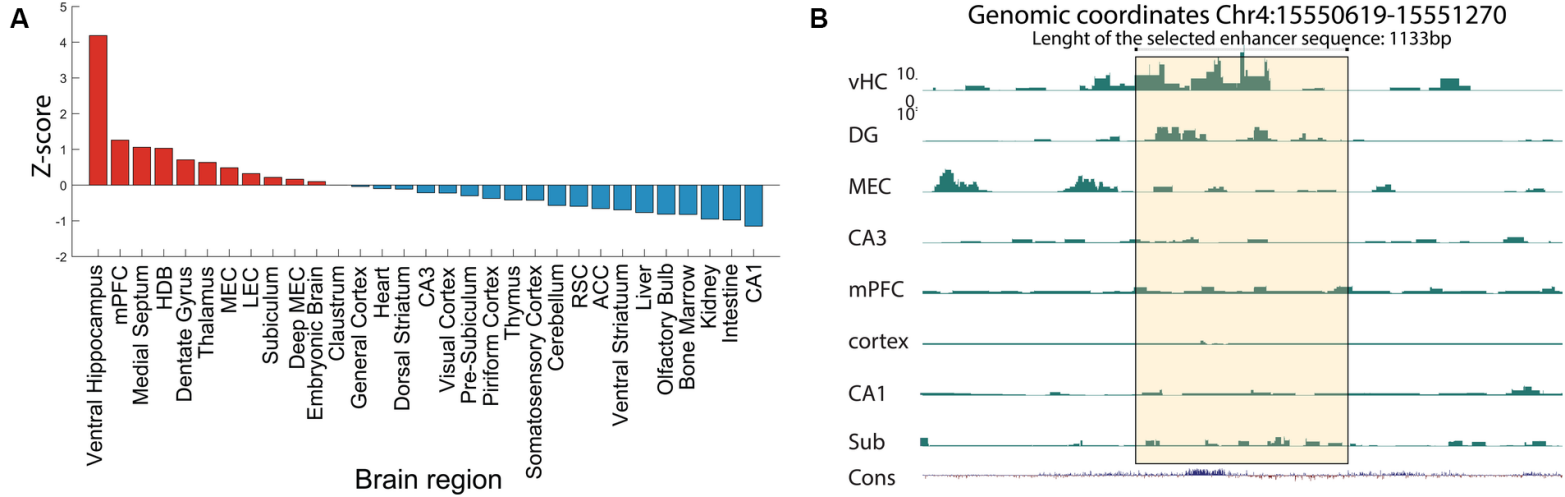
ChIP-seq reveals a novel enhancer in ventral hippocampus. **(A)** Example ChiP-seq data from the putative enhancer vHC-20-72, at genomic coordinates Chr4:15550619–15,551,270, extracted from ventral hippocampus tissue. The plotted data show relative activity of the enhancer compared to the reference signal. Note the high value for the ventral hippocampus, but also related regions such as the dentate gyrus and the medial prefrontal cortex. **(B)** H3K27Ac signal of the vHC-20-72 enhancer in a selection of tissue samples. The yellow shading indicates the vHC-20-72 enhancer region. The panel shows a zoomed in view. Bottom tracks show relative evolutionary conservation. Note the strong H3K27Ac signal for the ventral hippocampus, and to some extend the dentate gyrus, subiculum and MEC, but absence of signal in other regions. All Y-axis ranges are the same.

Strategies for investigating transcriptional control are crucial for creating cell-type specific tools in neuroscience. Toward this end we previously used *Chromatin Immunoprecipitation followed by high-throughput sequencing* (ChIP-seq) of four closely related cortical regions to create cell-type specific tools for the entorhinal and cingulate cortices (Blankvoort et al., 2018). In the present study we analyzed micro-dissected tissue from a new set of 19 other brain regions (Figures 1–3, raw data available from the GEO repository). To assess the activity of enhancers within these regions, we performed ChIP-seq targeting H3K27Ac, a robust marker of enhancer activation. By computing pairwise correlations of the genome-wide ChIP-seq signal, we could use hierarchical clustering to sort brain regions based on genetic similarity (Figure 2A). To identify enhancers likely to have tissue specific function we intersected subregion specific clusters of enhancers with peak-calls from other tissue types. The signal was then *z*-scored to filter out putative enhancers with a significantly unique tissue expression (Figure 2B). Clustering analysis shows that samples predominantly clustered together within their respective replicates. When considering broader groups in the cluster dendrogram, a biologically meaningful pattern emerged, with hippocampal regions grouping together and cortical regions forming a distinct cluster separate from non-cortical regions (Figure 2A). After peak calling on the individual samples, we generated a unified list of 150,289 putative enhancers. Relative activity of all enhancers identified is shown on the heatmap (Figure 2B).

From the tissue specific lists of unique putative enhancers, we selected several enhancers to test their cell-type specificity with the use of viral vectors. This selection was based on several criteria, the first of which is the *z*-score (Figure 2B) followed by the absence of repeat elements in the selected enhancer sequence. In fact, repetitive elements can introduce complexity and variability in the sequence, while we seek to identify unique and non-repetitive sequence that are more likely to have a specific functional role. The third criterion is the evolutionary conservation of the enhancer sequences that implies that the enhancer might play a crucial role in the regulation of genes or other biological functions if it has been preserved through natural selection. The most relevant enhancers are those sequences with high conservation between human and mice, as well as low degree of repeated elements. Finally, using *in situ hybridization* (ISH) data from the Allen Brain Atlas,^6^ we evaluated the expression patterns of the nearest genes, as the proximity of an enhancer to a gene might indicate a potential regulation of the gene expression. Of particular interest was a putative enhancer with a strong signal in the ventral hippocampus (vHC-20-72; Figure 3A). Besides a strong signal in the vHC, this putative enhancer had a strong signal in, amongst other regions, the medial prefrontal cortex (mPFC) and the dentate gyrus (DG), which was also clearly reflected in the raw signal (Figure 3B). The nearest gene to the putative enhancer was the one coding for calbindin-D28K (Calb-1), which suggests that this enhancer may aid the expression of this marker in distinct neuronal cell-types (Genomic coordinates Chr4: 15550619–15,551,270 https://genome.ucsc.edu/).

### Enhancer vHC-20-72 drives transgene expression in dentate granule cells in wild-type mice

After identifying eligible putative enhancers enriched in the hippocampus, we constructed rAAVs that could be stereotaxically injected. To accomplish this, we cloned putative enhancers in a viral vector backbone previously described by our group (Nair et al., 2020). Specifically, we used rAAV 2/1 (i.e., a chimera between capsids 1 and 2) which shows strong tropism for neurons (Davidson et al., 2000; During et al., 2003; Aschauer et al., 2013).

**Figure 4:**
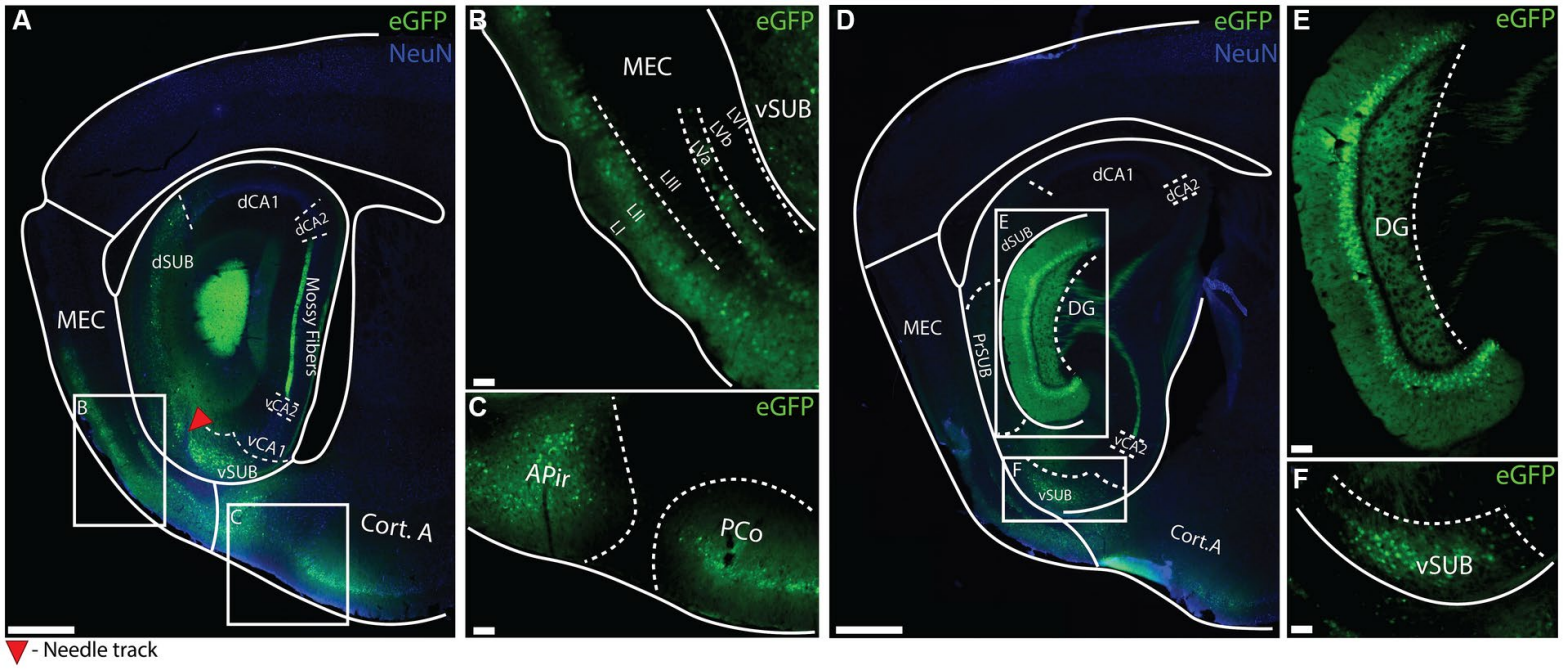
Expression of vHC-20-72 enhancer after a large-volume injection of EDGE rAAV 2/1 vHC-20-72 in the hippocampal formation of wild-type mouse. **(A–D)** Sagittal brain section showing eGFP (green) and NeuN (blue) expressing cells in wild-type mouse injected with EDGE rAAV 2/1 vHC-20-72-eGFP. The white boxes in **(A,D)** indicate the position of the higher magnifications shown in **(B–F)**. **(B)** High-magnification view of eGFP positive cells in medial entorhinal cortex (mainly in layer I, II and Va of MEC). **(C)** High- magnification view of eGFP positive cells in amygdala. **(E)** High magnification view of eGFP positive cells in dentate gyrus. **(F)** High magnification view of eGFP positive cells in ventral subiculum. Scale bar measures 500 μm in **(A)** and **(D)**, and 100 μm in insets.

To investigate whether the putative enhancer drives regionally specific transgene expression and to get an initial overview of which cell-types can be targeted, we performed a large volume stereotaxic injection (1,000 nL) of the EDGE rAAVs into the hippocampal region of C57BL6/J mice (Supplementary Table S1.A). This is about 10 times larger than typical stereotaxic injections, normally around 50-200 nL, allowing us to study the expression of the EDGE viruses in a large portion of the brain. The expression patterns were assessed 2 weeks post-injection. We tested several putative enhancers (Supplementary Table S2) but most of them did not appear to yield cell type specific expression (Supplementary Figure S1), the exception being the vHC-20-72 enhancer (Figure 4). Despite covering such a large area with the large volume injection (1,000 nL) of rAAV 2/1-vHC-20-72-eGFP, we found that the virus seemingly only infected specific cell populations, located in a few brain regions including the granule layer of the DG. Interestingly, we did not see any labeling in mossy cells in the hilus, indicating that only a sub-population of DG neurons were targeted (Figures 4D-E).

**Figure 5:**
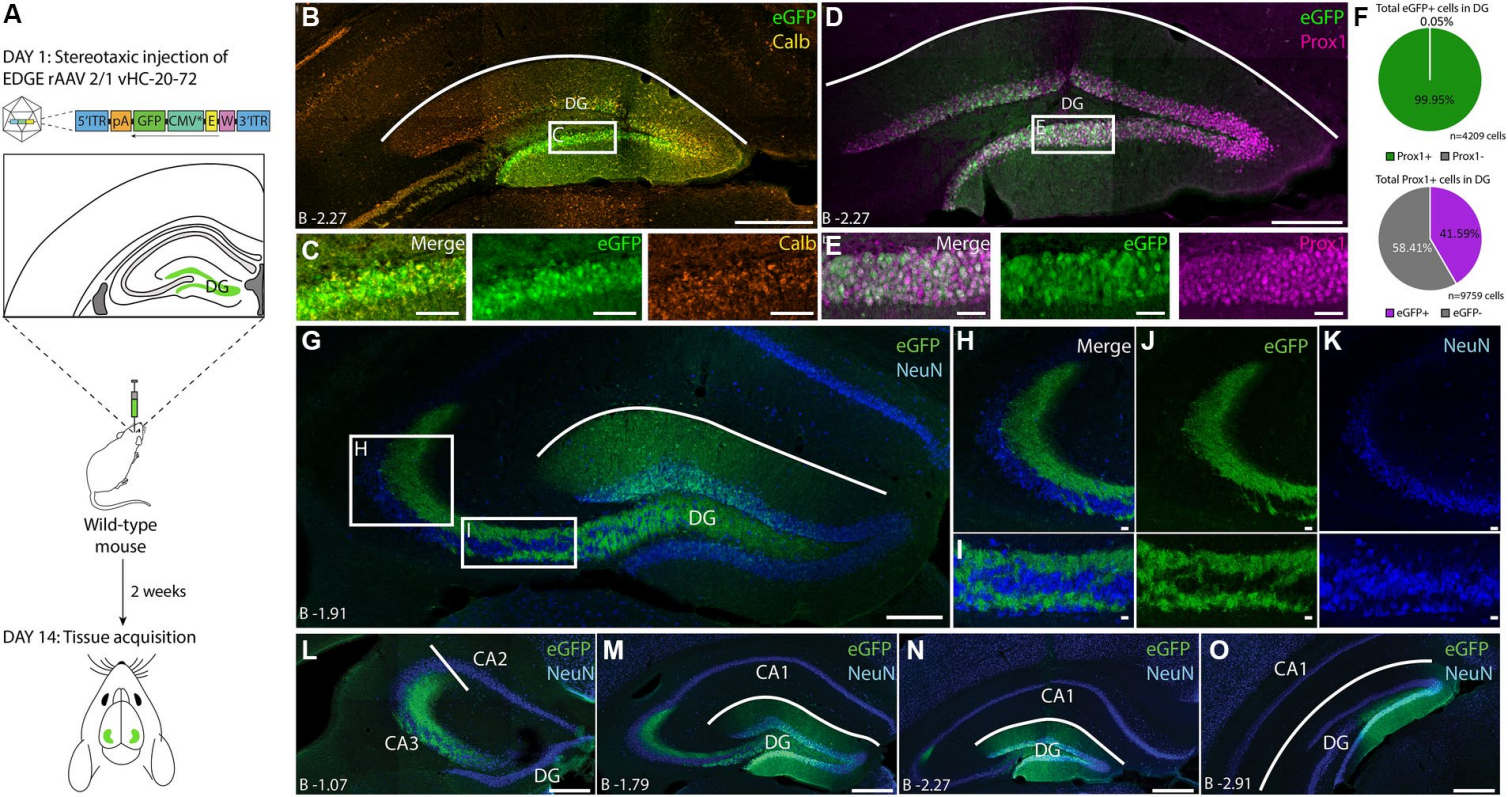
Expression of vHC-20-72 enhancer using a 2/1 rAAV serotype in dentate gyrus in wild-type mouse. **(A)** Schematic representation of the experimental design. A wild-type mouse is stereotaxically injected in dentate gyrus (DG) with 100 nL of EDGE rAAV 2/1 vHC-20-72-eGFP (10^12^ viral genomic particles/ml) and perfused after 2 weeks for tissue acquisition and immunohistochemistry analysis. **(B)** Coronal brain section showing eGFP (green) and Calb (orange) expressing cells in the DG. Note the extensive co-localization of the Calb staining with the eGFP cells (yellow overlap signal). Scale bar measures 500 μm. The white box in **(B)** indicates the position of the higher magnification shown in **(C)**. **(C)** High magnification view of co-expression of eGFP and Calb (left), eGFP expressing cells (middle) and Calb expressing cells (right). Scale bars measure 100 μm. **(D)** Coronal brain section showing eGFP (green) and Prox1 (magenta) expressing cells in the DG. Note the extensive co-localization of the Prox1 staining with the eGFP cells (white overlap signal). Scale bar measures 500 μm. The white box in **(D)** indicates the position of the higher magnification shown in **(E)**. **(E)** High magnification view of co-expression of eGFP and Prox1 (left), eGFP expressing cells (middle) and Prox1 expressing cells (right). Scale bars measure 100 μm. **(F)** Pie charts showing the percentage of all eGFP cells that co-expressed Prox1(green), and the percentage of all counted Prox1 cells that were also GFP positive (purple). **(G–O)** Immunohistochemical staining against eGFP (green) and NeuN (blue). **(G)** Overview image of DG showing eGFP expressing cells only in the granule layer of DG. Scale bar measures 500 μm. The white boxes in **(G)** indicate the positions of images shown in **(H)** and **(I)**. **(H-I)** High-magnification view of mossy fibers of DG. eGFP signal is present in the mossy fibers but not in the hilar mossy cells of DG. Scale bars measure 50 μm. **(L–O)**. Expression of eGFP (green) and NeuN (blue) in DG at 4 anterior–posterior landmarks. Approximate location to bregma is shown at each landmark. Scale bars measure 100 μm.

To test whether we could use this tool to precisely target dentate granule cells (DGCs), we performed smaller injections (100 nL), bilaterally in the DG (Figure 5A) and found that this was indeed the case (Figures 5B–D). To identify which cell-types were labeled we stained against the Calb-1 protein, which is a marker for DGCs, but since this gave a poor resolution for cell counting in the DG we changed to the DGCs-specific PROX1 marker instead (Figures 5B–E; Pleasure et al., 2000, Navarro-Quiroga et al., 2007). We found that cells labeled by the EDGE virus rAAV2/1 vHC-20-72-eGFP were almost exclusively PROX1 positive (99.95% overlap, Figure 5F). Our injections did not label all granule cells in the DG (41.59% of all PROX1+ cells were eGFP+), likely due to the virus not spreading through the entire DG, which typically requires multiple injections. This was also apparent as the granule cells in the direct vicinity of the injection site were eGFP+, whereas those further away showed a lower percentage of co-expression. Interestingly, the spread along the longitudinal axis is much further than along the transverse axis in the DG. As expected, we saw dense labeling of the mossy fibers that arise from DG granule cells, throughout the longitudinal axis of the hippocampus (Figures 5G–O). Other cell-types in the DG, including interneurons and hilar mossy cells, did not show any eGFP labeling, and there was also no labeling nearby in the *cornu ammonis* 3 (CA3) region (Figures 5G–I) Together, this shows that the rAAV 2/1-vHC-20-72-eGFP is a useful tool to target the granule cells in the DG.

**Figure 6:**
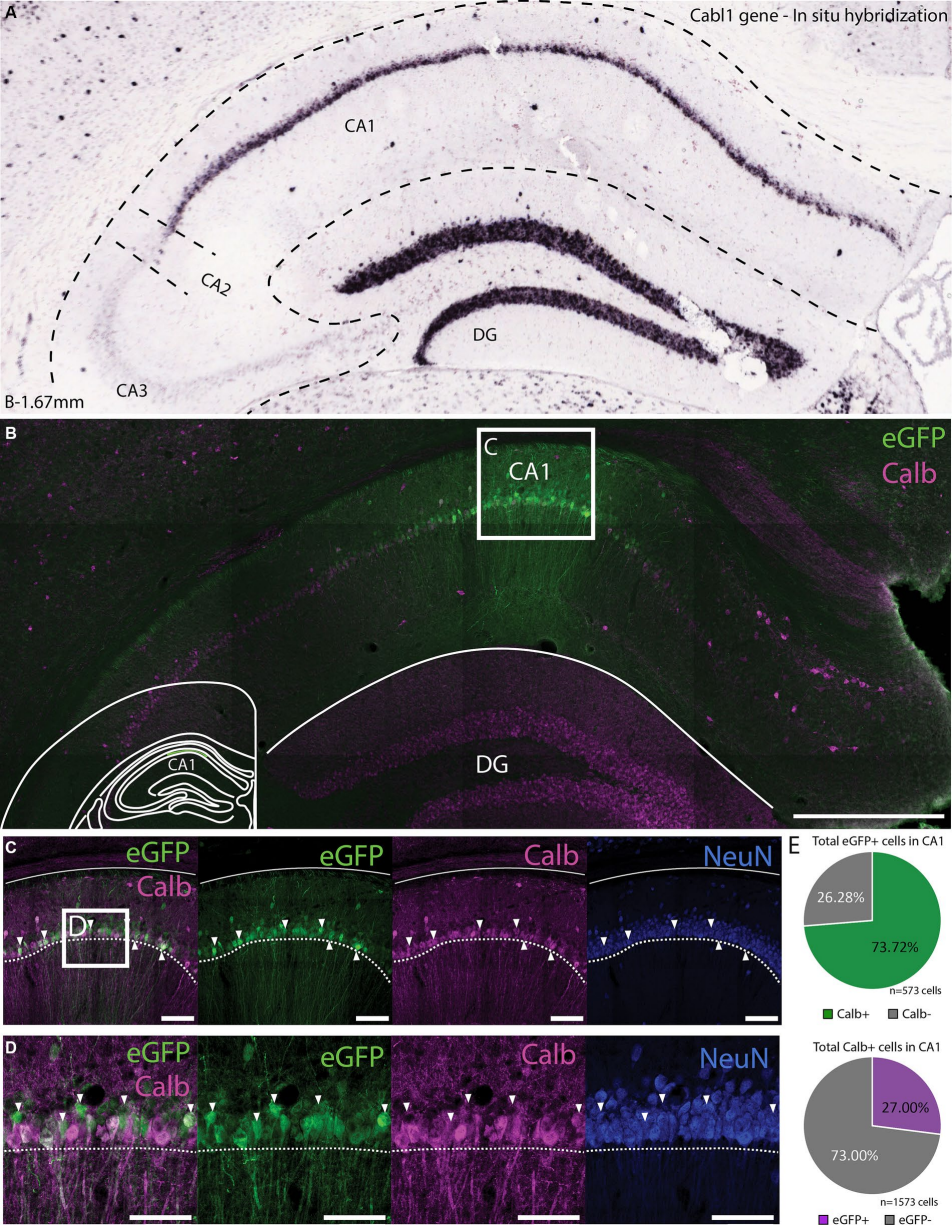
Expression of vHC-20-72 enhancer-based virus in dorsal CA1 in wild-type mouse. **(A)** *In situ hybridization* (ISH) labeling of the calbindin-D28k gene (Calb) in the dentate gyrus and CA1 region of the hippocampus. Image credit, Allen Brain Institute (https://mouse.brain-map.org/experiment/show/71717640). **(B)** Coronal brain section showing eGFP (green) and calbindin (magenta) expressing cells in a mouse injected with EDGE rAAV 2/1 vHC-20-72-eGFP in dorsal CA1. eGFP expressing cells in CA1 partially co-localize with calbindin positive cells. Scale bar measures 500 μm. The white box in (B) indicates the position of the higher magnification shown in **(C)**. **(C)** High magnification view of co-expression of eGFP and Calb. Scale bars measure 100 μm. **(D)** The same as C, with an even higher magnification. **(E)** Pie charts showing the percentage of all eGFP cells that co-expressed Calb (green), and the percentage of all counted Calb cells that were also GFP positive (purple).

Since the transgene expression in DG was specific to Calb expressing cells, as seen also by PROX1 labeling, we wanted to see if this was the case in other brain regions as well. A prime candidate to test this was the CA1 of the hippocampus due to the fact that this region contains numerous calbindin expressing cells (Figure 6A). Even though the ChIP-seq signal for CA1 was particularly low, injections of rAAV-vHC-20-72 in CA1 largely labeled cells in the pyramidal layer (Figures 6B–D), the majority of which co-expressed Calb (71.89% of all eGFP+ cells were Calb+; Figure 6E upper panel). Our injections did not label all Calb+ cells in the CA1, in part due to the spread of the virus but also among cells at the injection site (27.00% of all Calb+ cells in CA1 were labeled by eGFP; Figure 6E lower panel). In some cases where the injection landed too deep, we observed labeling in both CA1 and DG, warranting some care in how this tool is used to target these areas.

**Figure 7:**
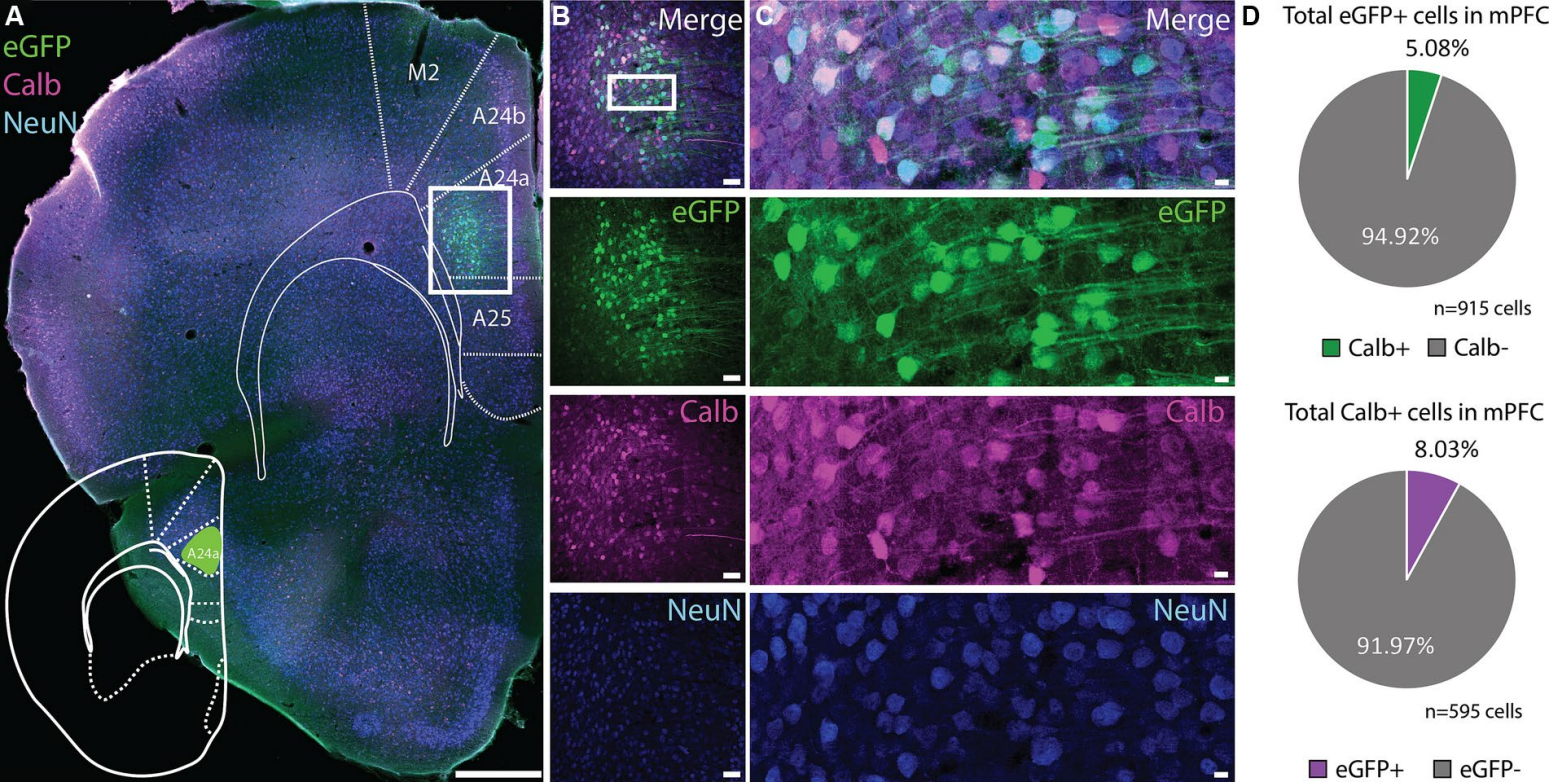
Expression of vHC-20-72 enhancer using a 2/1 rAAV serotype in medial prefrontal cortex in wild-type mouse. **(A)** Coronal brain section showing eGFP (green) and calbindin (magenta) expressing cells in a mouse injected with EDGE rAAV 2/1 vHC-20-72-eGFP in medial prefrontal cortex (mPFC). eGFP expressing cells in mPFC partially co-localize with calbindin marker. Scale bar measures 500 μm. (B) The white box in **(A)** indicates the position of the higher magnification shown in **(B)**. **(C)** High magnification view of co-expression of eGFP, Calbindin and NeuN. Scale bars measure 100 μm. **(D)** Pie charts showing the percentage of the eGFP population in mPFC that co-expressed calbindin (green), and the percentage of the total calbindin population that co-expressed eGFP (purple).

Based on the z-score signal for the vHC-20-72 enhancer another good candidate would be the mPFC. To our surprise, the expression of rAAV 2/1-vHC-20-72-eGFP in the mPFC was not confined to Calb positive cells (Figures 7A–C). Rather, only a small fraction of eGFP+ cells co-expressed Calb (5.08%, Figure 7D upper panel). Calb cells were highly abundant in this area, and eGFP labeled cells were almost complementary to this population (8.03% of all Calb cells in mPFC expressed eGFP, Figure 7D lower panel). Similarly, when injecting this virus into the dorsal subiculum there was no overlap between eGFP labeled cells and Calb (Supplementary Figure S2). Thus, it seems that the genetic selectivity of this tool is not directly correlated to the Calb gene, although it does label this population with high precision in some brain regions. To identify the labeled cell populations in non-DG regions, we looked at different genetic marker candidates that have been used to characterize mPFC and subiculum cell-types (Cembrowski et al., 2018; Bhattacherjee et al., 2019), and compared expression patterns of these genes using the Allen Brain *In Situ* Hybridization database, to the labeling we saw in our injection data (Supplementary Figure S3). We found that candidate markers such as *Sema6d*, *Tpgb*, and *S100b* aligns well with the location of targeted cells we saw in subiculum, whereas *Foxp2*, *Syt6* and *Sema5a* aligned with the population in mPFC.

### Edge rAAV 2/1 vHC-20-72 transgene expression in early postnatal development

**Figure 8:**
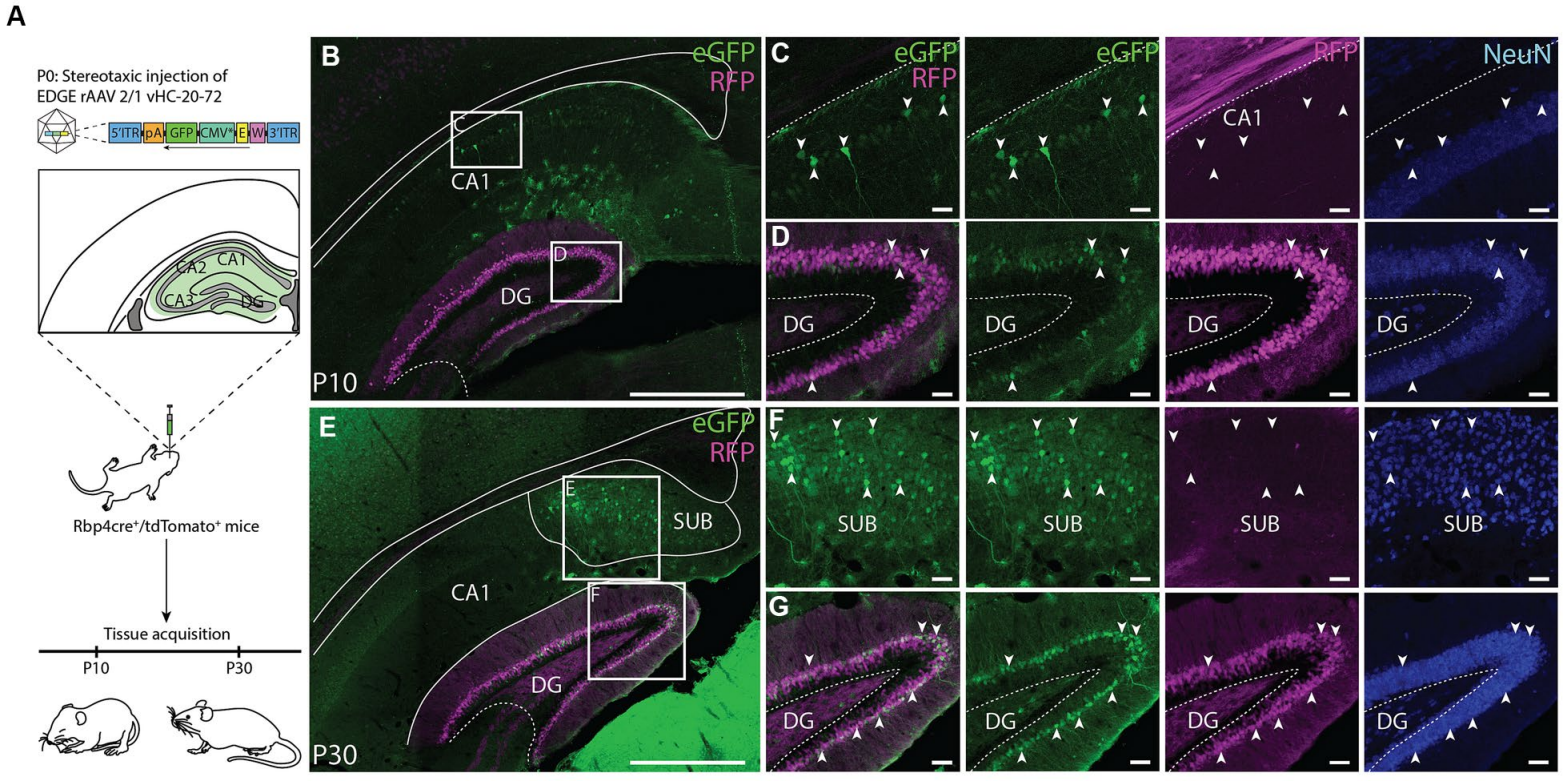
Application of the EDGE rAAV 2/1 vHC-20-72 at early post-natal development. **(A)** Schematic representation of the experimental design. Rbp4-cre+/tdTomato+ mice were stereotaxically injected in the hippocampal formation at post-natal day 0 (P0) with EDGE rAAV 2/1 vHC-20-72-eGFP (10^12^ viral genomic particles/ml) and perfused at two different time-points (P10 and P30) for tissue acquisition and immunohistochemical analysis. **(B–E)** Coronal brain section showing eGFP (green) and RFP (magenta) expressing cells at P10 and P30, respectively. Scale bar measures 500 μm. **(D–G)** High-magnification view of eGFP (green) and RFP (magenta) expressing cells in DG. **(C–F)** High-magnification view of eGFP (green) and RFP (magenta) expressing cells in CA1 and Subiculum, respectively. Scale bars measure 100 μm.

To further investigate the utility of the rAAV2/1 vHC-20-72-eGFP to study the DG, we used the Rbp4-Cre+/tdTomato+ transgenic mice, in which DGCs are specifically labeled (Gerfen et al., 2013; Egger et al., 2023). Since enhancer elements play a crucial role in the spatio-temporal orchestration of gene expression during development (Pachano et al., 2022), we aimed to determine the viability of transgene expression at early postnatal age. We thus carried out an experiment by injecting the EDGE rAAV at post-natal day 0 (P0) and euthanizing the mice at two different time points: P10 and P30 (Figure 8A). After the stereotaxic injection, which targeted cells along the entire hippocampus - we found expression primarily in DGCs, both at P10 and P30 (Figures 8B,E). Interestingly, labeling was only seen in a subset of DG cells that were found in a laminar fashion bordering the molecular layer (Figure 8G). The reason for this laminar subset of labeling may be that infection occurred in cells generated around the moment of stereotaxic injection. Unlabeled cells may therefore have appeared after the injection due to post-natal neurogenesis (Amaral et al., 2007). Additionally, the expression seemed to occur along the entire longitudinal axis as well as the entire transverse axis.

**Figure 9:**
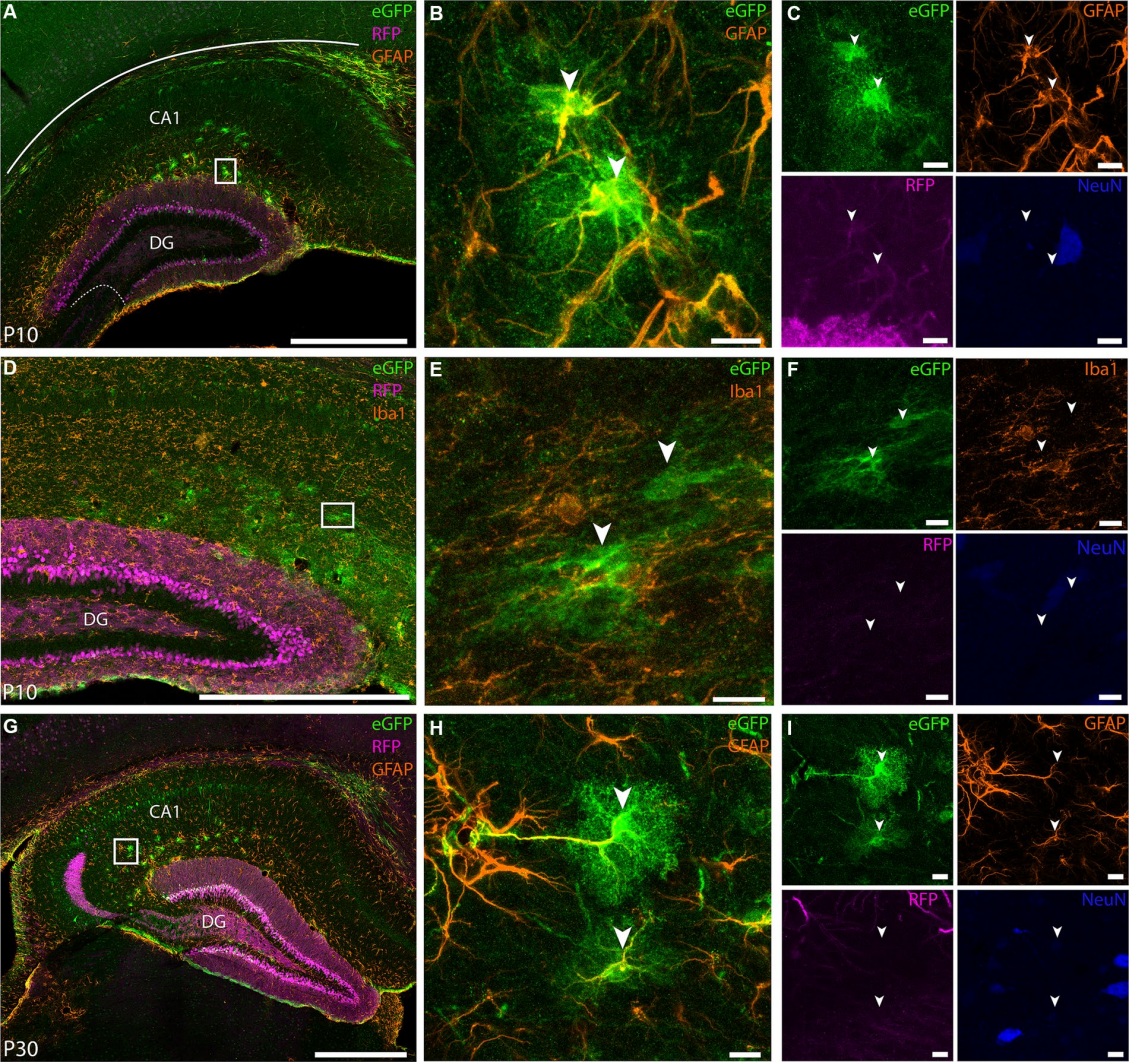
EDGE rAAV 2/1 vHC-20-72 labels astrocytes at early post-natal development. Co-expression of the EDGE virus with GFAP marker in mouse pups. White boxes indicate the position of the higher magnification images, with panels **(B,C)** corresponding to **(A)**, **(E,F)** to **(D)** and **(H,I)** to **(G)**. **(A)** Coronal brain section showing eGFP (green), RFP (magenta) and GFAP (orange) expressing cells at P10. **(B)** High-magnification view of co-expression of eGFP and GFAP (yellow overlap signal) at P10. **(C)** Same as in **(B)** but with individual expression of eGFP (green), GFAP (orange), RFP (magenta), and NeuN (blue). **(D)** Coronal brain sections showing eGFP (green), RFP (magenta) and Iba1 (orange) expressing cells at P10. **(E–F)** High-magnification view of eGFP (green), Iba1 (orange), RFP (magenta), NeuN (blue) expressing cells. Note the absence of overlap between eGFP+ cells and Iba1. **(G)** Coronal brain sections showing eGFP (green), RFP (magenta) and GFAP (orange) expressing cells at P30. **(H–I)** same as **(B-C)** but for the inset shown in **(G)**. Scale bars measure 500 μm in **(A)**, **(D)**, and **(G)** and 100 μm in insets.

Furthermore, based on the expression of the Calb gene in online material from the Allen Brain Institute^7^, at P4 this gene is not uniformly present but confined to a subset of DGCs in the inner blade, whereas at P14, the expression becomes widespread. This observation is in line with the notion that the virus maintains specificity toward DGCs, even though the enhancer is not active at the time of injection (P0). Notably, at P30, a substantial increase in the number of cells expressing Calb is evident. In addition to labeling in the DG, we found eGFP+ cells in CA1 and subiculum (Figures 8C,F), corroborating the expression pattern we saw in adult animals. Surprisingly, in pup brains we also observed some labeling in astrocytes, which is something that was never observed when this viral tool was used in adult animals (Figure 9). We used GFAP (Figures 9A–G) and Iba1 (Figure 9D) markers to evaluate whether eGFP+ cells were astrocytes or microglia, respectively. We found co-expression between eGFP and GFAP both at P10 and P30 (Figures 9B,C,H) and no overlap between eGFP and Iba1 (Figures 9E,F). Overall, these results confirm the usefulness of this viral tool to study DGCs, but also highlights that some care should be taken with regards to the injection strategy if only granule cells are to be studied in pups.

### Cross-species applicability of EDGE rAAV 2/1 vHC-20-72 for transgene expression in dentate granule cells in rat

**Figure 10:**
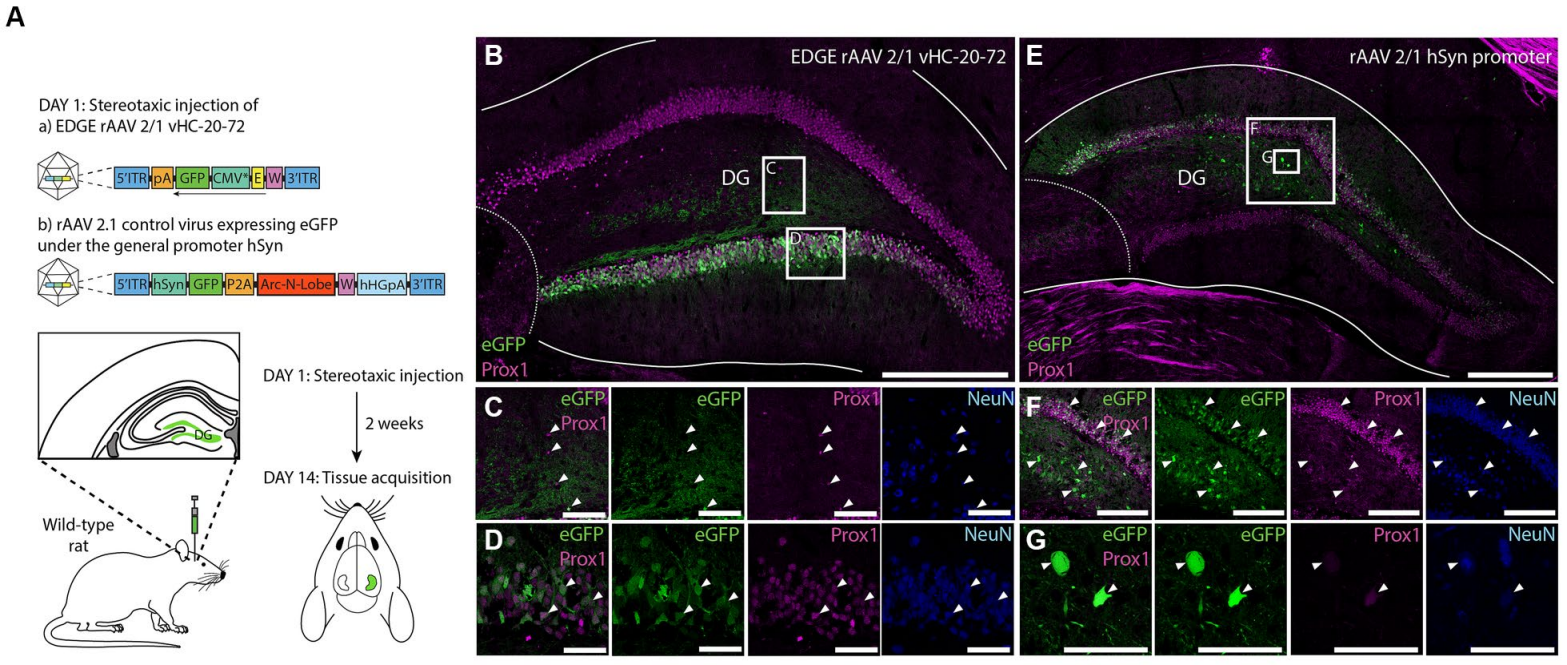
Expression of vHC-20-72 enhancer using a 2/1 rAAV serotype in dentate gyrus in wild-type rat. **(A)** Schematic representation of the experimental design. Sprague Dawley rats were stereotaxically injected in DG either with 1,000 nL of EDGE rAAV 2/1 vHC-20-72-eGFP (10^12^ viral genomic particles/ml), or with 1,000 nL of a control virus rAAV 2/1 hSyn-eGFP (10^11^ viral genomic particles/ml). Two weeks after the viral injections, rats are perfused for tissue acquisition and immunohistochemistry analysis. (B) Coronal brain section showing eGFP (green) and Prox1 (magenta) expressing cells in rat DG, injected with EDGE rAAV 2/1 vHC-20-72-eGFP. The white boxes in **(B)** indicates the position of the higher magnification shown in **(C)** and **(D)**. **(C–D)** High-magnification view of co-expression of eGFP and Prox1. **(E)** Coronal brain section showing eGFP (green) and Prox1 (magenta) expressing cells in DG injected with control virus rAAV 2/1 expressing eGFP under the general promoter hSyn. Note the eGFP expressing cells both in the hilus and granule layer of DG. The red boxes in **(E)** indicates the position of the higher magnification shown in (F) and (G). (F-G) High-magnification view of co-expression of eGFP and Prox1. Scale bars measure 500 μm in (B) and (E), and 50 μm in insets.

Given that enhancers elements have highly conserved function (Cotney et al., 2013; Wong et al., 2020), we next investigated cross-species applicability of our viral tool, and examined whether the enhancer vHC-20-72 can drive transgene expression in dentate granule cells of rats (Figure 10A). After stereotaxic injection of rAAV 2/1-vHC-20-72-eGFP to the DG of rat, we found that expression of the transgene under control of the selected enhancer was specific for the granule cells in the DG (Figures 10B–D), confirming the results previously obtained in wild type mice and Rbp4-Cre+/tdTomato+ mice. To control for limited spread of the viral vector, we injected a control virus rAAV 2/1 with a transgene under control of the universal human synapsin promoter (hSyn, Figures 10E–G). In this case, we found eGFP+ cells in both granule and hilar cells. Moreover, the EDGE virus showed a higher specificity even though it had 10 times higher titre than the control virus. All together, these results show that even though the viral vector infects cells throughout the DG, only the granule cells express the transgene under control of the vHC-20-72 enhancer. Considering the lack of transgenic lines for the study of DG in rats, this viral tool holds the promise of providing experimental access to dentate granule cells.

### An expansion of the EDGE rAAV toolkit

In the present study, we expand upon the available toolkit for EDGE technology by performing ChIP-seq on 19 microdissected subregions of the mouse brain and differentially screening for putatively unique enhancers, in this case targeting the hippocampal formation. We present experiments with rAAVs made from one of the resulting enhancers (vHC-20-72), which nicely illustrates both the capacity enhancers have to drive cell-type specific expression and the complexity of the epigenetic interactions that dictate gene expression in the brain. Our novel enhancer maps to the calbindin gene, and at least in the hippocampus appears to drive expression in Calb positive excitatory neurons (see Figures 5, 6) in both wildtype mice and rats (Figure 10). However, in both the subiculum and infralimbic cortex, brain regions rich in Calb-positive neurons, the virus quite specifically seems to *exclude* neurons expressing this marker. Regardless, our experiments demonstrate that this is still a useful tool for dissecting out the roles of distinct cell-types, although some surgical precision is needed to attain this goal.

## Discussion

In the present study we describe the process of generating enhancer-based rAAVs specific to neuronal cell-types and characterize a novel EDGE rAAV, which illustrates how such tools can be created and used. We also share a database of ChIP-seq hits in subregions of mouse brain. In general, while EDGE-rAAVs are far more specific than promoter-based tools, this does not mean that they exclusively express only in a single cell-type. Furthermore, while an enhancer may reliably drive expression in particular neuronal cell-types, it acquires this specificity through a combination of mechanisms. For example, most enhancers interact with complexes of transcription factors, which are highly numerous in the brain (Vaquerizas et al., 2009; Long et al., 2016; Lambert et al., 2018). Moreover, enhancers have been shown to interact with more than one such transcription factor complex *in vitro* (Kyrchanova and Georgiev, 2021), so it is not entirely surprising that they express in several different neuronal cell-types. The heightened specificity seen in the transgenics made with the same enhancer may indicate that epigenetic changes during neurodevelopment plays a significant role in the specification of neuronal cell-types, an interesting avenue of future research.

However, these epigenetic mechanisms do not occur when an EDGE-rAAV is injected in a wild-type animal, which might explain why we obtained sparse labeling in multiple distinct regions in our experiments. While we can only speculate on the exact regulatory underpinnings that determine the expression of an enhancer based viral tool, it is often assumed that a given enhancer acts upon the most proximal gene. The vHC-20-72 enhancer characterized in this study was found close to the calbindin-D28k (Calb) gene, and although it produced specific labeling of Calb-positive cells in the DG, it only labeled Calb-negative cells in the subiculum. As such, it seems that whatever genetic regulation is enforced by the vHC-20-72 enhancer, is not only related to the presence of Calb. This somewhat puzzling result suggests a potential explanation for the higher promiscuity of EDGE rAAVs relative to transgenic animals made with the same enhancer. There are several distinct Calb+ cell-types (DeFelipe, 1997; Gonchar and Burkhalter, 1997), and while the Calb promoter of course drives expression in them all, this particular enhancer may be primarily involved in expressing Calb in the hippocampus, but not in other brain regions. The label in Calb-cells outside of the hippocampus proper, however, may be because they express transcription factors that can interact with the enhancer in the AAV, while the native enhancer is epigenetically silenced in these cells. Although in some cases this is a limitation to EDGE, in cases where more than one relevant brain region is cleanly targetable with stereotactic injections, this might become an advantage, provided that the regions in question are far enough apart. Such is the case for the EDGE transgenic line MEC-13-53D, which has been used to achieve highly specific targeting both in deep layers of the entorhinal cortex and the claustrum complex (Blankvoort et al., 2018; Ohara et al., 2021; Grimstvedt et al., 2023). Thus, it is crucial to characterize the brain-wide expression pattern of EDGE tools, to determine the distance between different targetable areas.

Even if EDGE-based viruses might be susceptible to off-target expression, they still offer the unique opportunity to conduct cell-type specific investigations in wild-type animal, which require less time and resources than transgenic lines, and also circumvents potential issues of insertional effects (Feng et al., 2000; Matthaei, 2007). Moreover, one can use them to obtain cell-type specific expression also in the context of a transgenic line (e.g., disease models). Our results with vHC-20-72 led to specific labeling of DGCs in both wild type mice and rats. This is significant because rats are often used in memory research, where the DG plays a central role (Jessberger et al., 2009; Mendez-Couz et al., 2015; Fernandez-Ruiz et al., 2021) but there is only a very limited set of transgenic rat lines (Leon et al., 2010; Snyder et al., 2016). The vHC-20-72 enhancer could therefore be of great use to memory research with rats, a favored species for behavior and neurophysiology. Moreover, considering how enhancer activity is largely maintained across species, it is reasonable to assume that our viral tools could also be applicable to other rodent models.

We also studied the expression patterns at early post-natal stages, since age can highly influence enhancer activity due to variations in genetic regulation throughout development (Ma and Zhang, 2020; Zhang et al., 2022). During initial developmental phases there are massive changes in neuronal architecture, which is also reflected in the transcriptional regulation of gene expression (Mason et al., 2012; Harabula and Pombo, 2021; Janssen and Lorincz, 2022; Ramirez et al., 2022; Shirane, 2022). It is therefore important to assess enhancer expression patterns in animal of different ages. By injecting the viral construct carrying the vHC-20-72 enhancer in newborn pups, we confirmed that the expression pattern was indeed retained, although the virus did also show some astrocytic labeling that was not present in adult animals. This represents a caveat that should be considered for using this tool in developmental studies. Interestingly, the DG is one of the few brain regions with adult neurogenesis, which raises another intriguing possibility for how this viral tool can be used.

In our experience, relating genomic data to viral expression patterns is not entirely straightforward. Our results from both the ChIP-seq data and viral expression of the vHC-20-72 enhancer included many of the same regions, such as the vHC, DG, mPFC and subiculum. However, it is not necessarily the case that the assigned z-scores from a ChIP-seq analysis translates directly to how specific a given enhancer will be as a viral tool, as enhancer activity can exhibit considerable spatiotemporal variability that may not be accurately represented by the sampled tissue (Zinzen et al., 2009; Ye et al., 2020). Another limitation in our dataset is that not every region of the brain was represented among the micro-dissected tissue we analyzed, including the dorsal parts of the hippocampus, which was also labeled by the vHC-20-72 virus. Although we cannot directly assess this with our ChIP-seq data, we can infer some things based on known genetic marker patterns in the hippocampus, where a large portion of identified markers distribute along a dorsoventral gradient, whereas only a few, including *Slc17a7* and *Zbtb20*, express more generally (Nielsen et al., 2007; Cembrowski et al., 2016). As such, it may be that the vHC-20-72 enhancer is not involved in regulating gene expression specific to dorsoventral domains within the hippocampus.

Although EDGE rAAV tools do not exclusively target only one cell-type throughout the brain, they are still a lot more precise than promoter-based targeting strategies, and as we show in the present paper, can be used to achieve cell-type specific labeling when locally injected. In future studies we aim to use a systemic injection strategy, to attain an overview of the expression pattern driven by the enhancer on a brain wide scale. To this end, the PHP.eB serotype might be a good candidate, as it has been shown to have a highly efficient transduction across the blood brain barrier (BBB) (Huang et al., 2023), more so than its predecessor PHP.B (Deverman et al., 2016). The development of BBB crossing EDGE viruses might also be relevant for future clinical application in gene therapy research. Additionally, future EDGE viruses could also express many different payloads aside from just an eGFP tag, such as optogenetic actuators or calcium indicators, which is currently one of the major advancements that have yet to be accomplished in enhancer-based targeting strategies. Part of the challenge is to ensure a strong enough expression to be applicable for such purposes, while still maintaining high levels of specificity, which may require the inclusion of other molecular tools such as AAVs utilizing recombinase tet-transactivator systems. Such advancements have yet to be developed, and until then the main utility of these tools is in providing an easy to use and highly specific approach of tracing neuronal pathways. The generation of cell-type specific viral tools represents one of the major goals in neuroscience and requires an enormous amount of effort from the scientific community at large. Overall, we believe that the principles underlying EDGE technology will have a significant impact toward this goal, though the full potential of this approach requires its application on a much larger scale (Graybuck et al., 2021).

## Data availability statement

The datasets presented in this study can be found in online repositories. The names of the repository/repositories and accession number(s) can be found in the article/Supplementary material.

## Author contributions

MP: Conceptualization, Data curation, Formal analysis, Investigation, Visualization, Writing – original draft, Writing – review & editing. SB: Conceptualization, Data curation, Investigation, Methodology, Resources, Software, Supervision, Visualization, Writing – review & editing. MC: Conceptualization, Investigation, Supervision, Writing – review & editing. JG: Conceptualization, Data curation, Visualization, Writing – original draft, Writing – review & editing. VM: Conceptualization, Investigation, Supervision, Writing – review & editing. KM: Conceptualization, Data curation, Investigation, Writing – review & editing. RN: Conceptualization, Investigation, Methodology, Resources, Writing – review & editing. MF: Investigation, Writing – review & editing. QZ: Investigation, Writing – review & editing. LT: Data curation, Formal analysis, Resources, Software, Writing – review & editing. FP: Data curation, Investigation, Writing – review & editing. RS: Writing – review & editing, Investigation. GQ: Conceptualization, Funding acquisition, Project administration, Resources, Supervision, Writing – review & editing. CB: Conceptualization, Funding acquisition, Project administration, Resources, Supervision, Writing – review & editing. PS: Writing – review & editing. MW: Conceptualization, Funding acquisition, Project administration, Resources, Supervision, Writing – review & editing. CK: Conceptualization, Funding acquisition, Methodology, Project administration, Resources, Supervision, Writing – review & editing.

## Footnotes

^1^ https://www.jax.org/strain/000664

^2^ https://www.taconic.com/mouse-model/b6jbom

^3^ https://www.mmrrc.org/catalog/sds.php?mmrrc_id=37128

^4^ https://www.jax.org/strain/007909

^5^ https://www.criver.com/products-services/find-model/cd-sd-igs-rat?region=3616

^6^ https://mouse.brain-map.org/search/index

^7^ https://developingmouse.brain-map.org/
