# Supplementary figures and images for: Generation of an enhancer-driven gene expression viral tool specific to dentate granule cell-types through direct hippocampal injection
Source: Front Neurosci. 2024 Mar 14;18:1274174. doi: 10.3389/fnins.2024.1274174 (PMC10976853; doi:10.3389/fnins.2024.1274174)

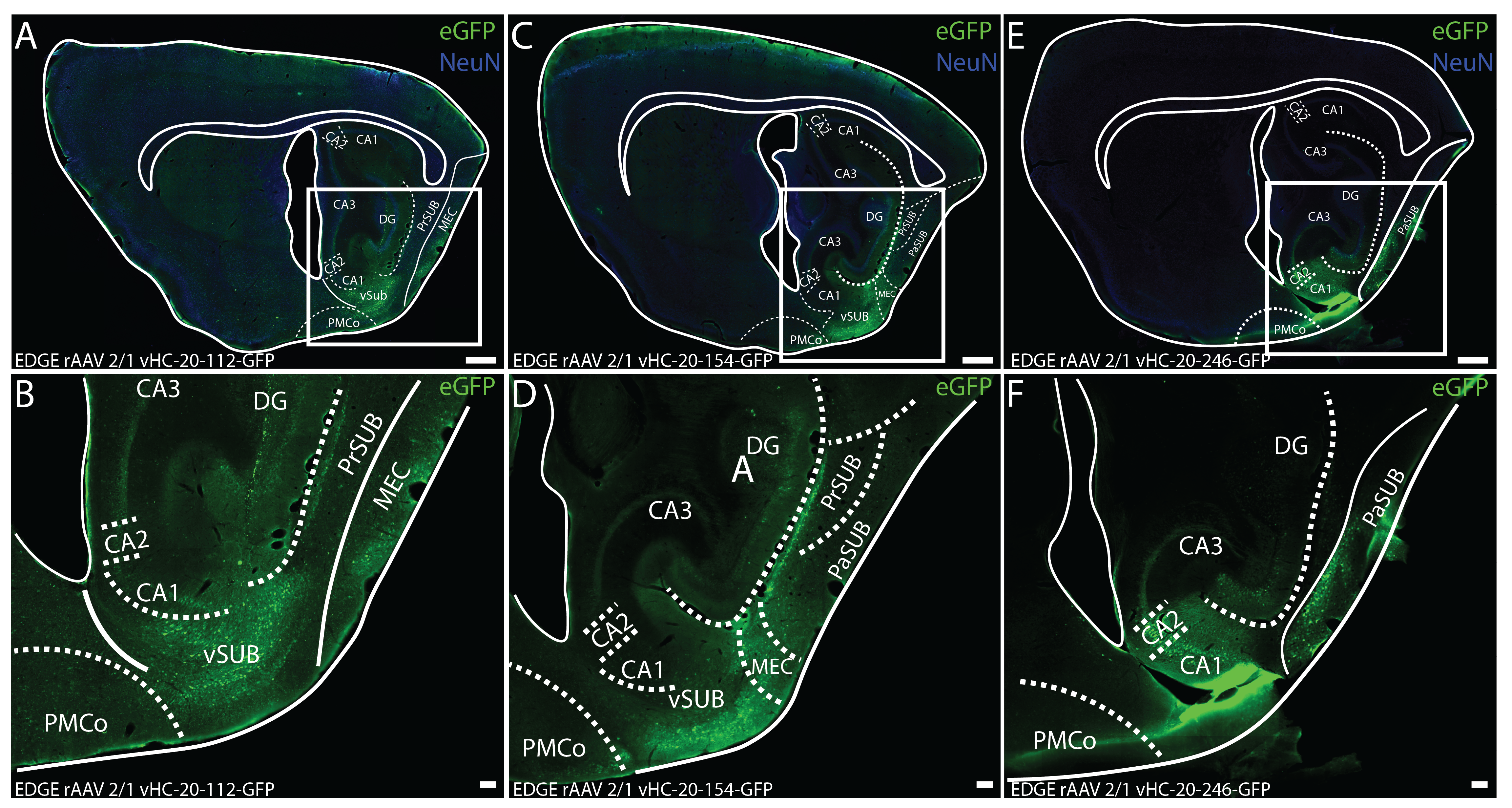

Supplement: Supplementary file 1 [file Image_1.TIFF]

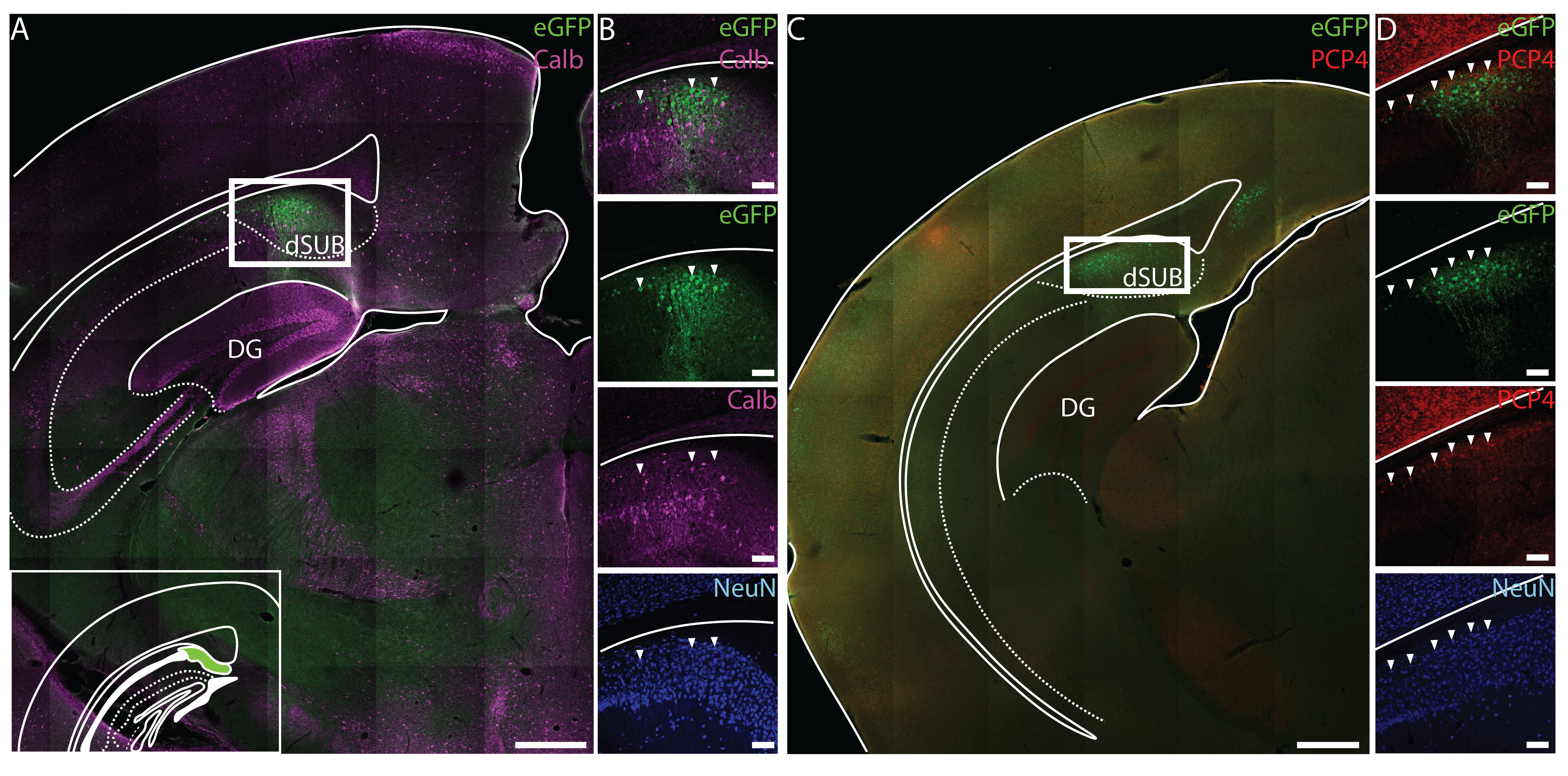

Supplement: Supplementary file 2 [file Image_2.TIFF]

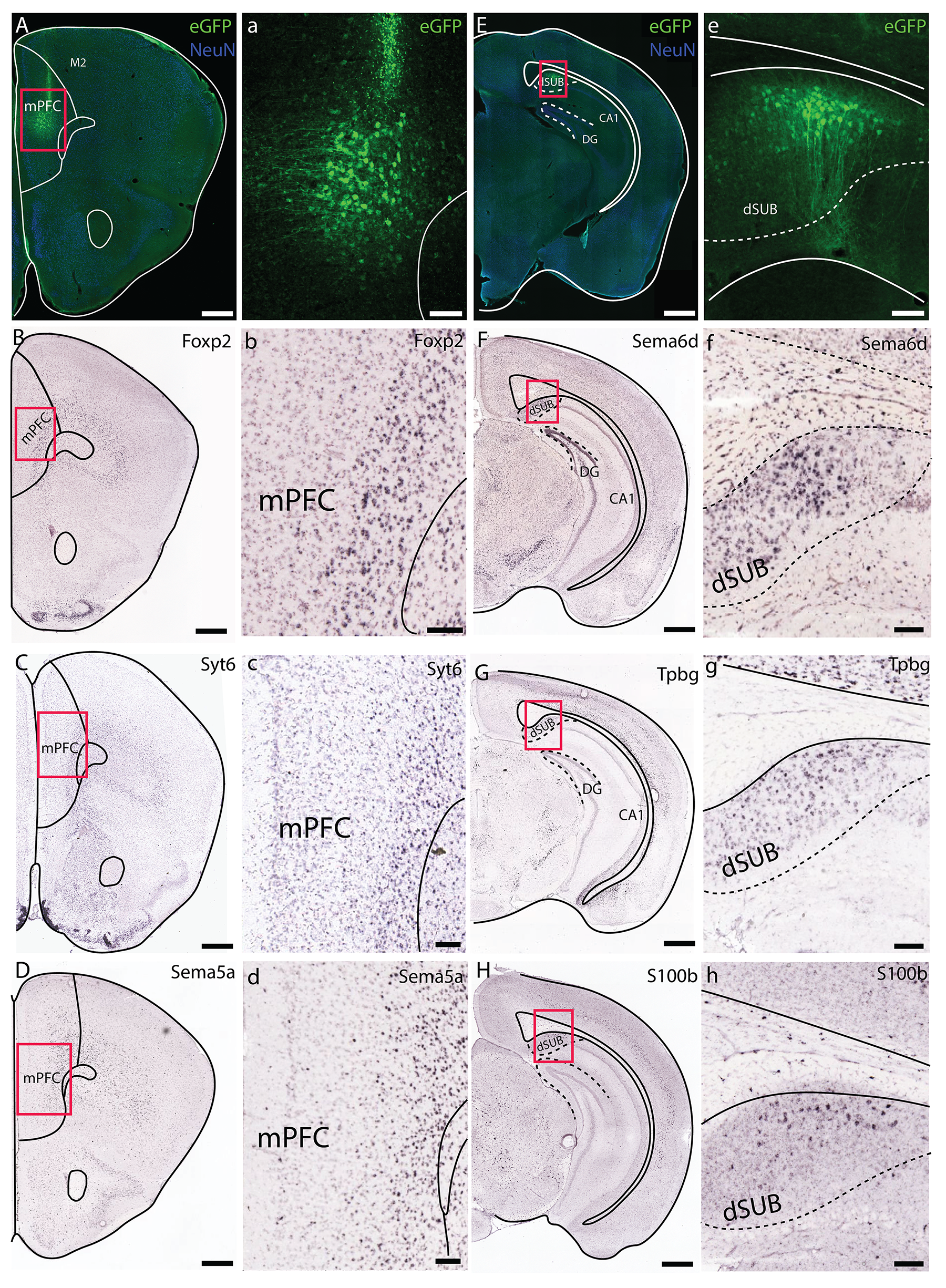

Supplement: Supplementary file 3 [file Image_3.TIF]
