# Supplementary material for: Generation of an enhancer-driven gene expression viral tool specific to dentate granule cell-types through direct hippocampal injection
Source: Front Neurosci. 2024 Mar 14;18:1274174. doi: 10.3389/fnins.2024.1274174 (PMC10976853; doi:10.3389/fnins.2024.1274174)
Supplement: Supplementary file 4 [file Table_1.DOCX]

| **Table S1.A: Massive stereotaxic injections in adult mice for enhancer screening (N=5)** | | | | | | |
| --- | --- | --- | --- | --- | --- | --- |
| **Animal ID** | **Breed** | **Virus** | **Injected at** | **Perfused** | **Unilateral injection** | **Target Brain region** |
| 97304 | C57BL6/J | rAAV 2/1 vHC-20-72-eGFP | 21w | 2 w.p.i. | 1000nl | HC formation |
| 97424 | C57BomTac: +/+ | rAAV 2/1 vHC-20-72-eGFP | 22w1d | 2 w.p.i. | 1000nl | HC formation |
| 97815 | C57BomTac: +/+ | rAAV 2/1 vHC-20-112-eGFP | 14w5d | 2 w.p.i. | 1000nl | HC formation |
| 97817 | C57BomTac: +/+ | rAAV 2/1 vHC-20-154-eGFP | 14w5d | 2 w.p.i. | 1000nl | HC formation |
| 97425 | C57BomTac: +/+ | rAAV 2/1 vHC-20-246-eGFP | 221d | 2 w.p.i. | 1000nl | HC formation |
| **Coordinates**  **AP**: -3.16mm; **ML:** +/- 3.1mm; **DV:** -4mm | | | **Viral titer**  rAAV 2/1 vHC-20-72-eGFP: 5.6x10^12^ viral genomic particles/ml  rAAV 2/1 vHC-20-72-eGFP: 1.5x10^12^ viral genomic particles/ml  rAAV 2/1 vHC-20-72-eGFP: 9.86x10^12^ viral genomic particles/ml  rAAV 2/1 vHC-20-72-eGFP:2.08x10^12^ viral genomic particles/ml | | | |

| **Table S1.B: Stereotaxic injections in adult mice (N=7)** | | | | | | | |
| --- | --- | --- | --- | --- | --- | --- | --- |
| **Animal ID** | **Breed** | **Virus** | **Injected at** | **Perfused** | **Left Hemisphere** | **Right Hemisphere** | **Target Brain region** |
| 99850 | C57BL6/J | rAAV 2/1 vHC-20-72-eGFP | 19w5d | 2 w.p.i. | 100nl | 100nl | mPFC and DG |
| 99851 | C57BL6/J | rAAV 2/1 vHC-20-72-eGFP | 19w6d | 2 w.p.i. | 100nl | 100nl | mPFC and DG |
| 99853 | C57BL6/J | rAAV 2/1 vHC-20-72-eGFP | 19w6d | 2 w.p.i. | 100nl | 100nl | mPFC and DG |
| 99856 | C57BL6/J | rAAV 2/1 vHC-20-72-eGFP | 19w6d | 2 w.p.i. | 100nl | 100nl | mPFC and DG |
| 66323 | C57BL6/J | rAAV 2/1 vHC-20-72-eGFP | 17w2d | 2 w.p.i. | 100nl | 100nl | dCA1 and dSub |
| 66324 | C57BL6/J | rAAV 2/1 vHC-20-72-eGFP | 17w2d | 2 w.p.i. | 100nl | 100nl | dCA1 and dSub |
| 66325 | C57BL6/J | rAAV 2/1 vHC-20-72-eGFP | 17w2d | 2 w.p.i. | 100nl | 100nl | dCA1 and dSub |
| **Coordinates**   - **mPFC:** **AP**: +1.9mm; **ML:** +/- 0.5mm; **DV:** -1.9mm - **dDG**: **AP**: -2.1mm; **ML:** +/- 1.5mm; **DV:** -2.2mm - **dCA1**: **AP**: -2mm; **ML:** +/-1.5mm; **DV:** -1.45mm - **dSUB**: **AP**: -3.4; **ML:** +/-1.8; **DV:** -1.4mm | | | **Viral titer**  rAAV 2/1 vHC-20-72-eGFP: 5.6x10^12^ viral genomic particles/ml | | | | |

| **Table S1.C: Stereotaxic injections in adult rats (N=8)** | | | | | | |
| --- | --- | --- | --- | --- | --- | --- |
| **Animal ID** | **Breed** | **Virus** | **Injected at** | **Perfused** | **Left Hemisphere** | **Target Brain region** |
| CBR04681 | Sprague Dawley | rAAV 2/1 vHC-20-72-eGFP | 2 months | 2 w.p.i. | 1000nl | DG |
| CBR04682 | Sprague Dawley | rAAV 2/1 vHC-20-72-eGFP | 2 months | 2 w.p.i. | 1000nl | DG |
| CBR04671 | Sprague Dawley | rAAV 2/1 vHC-20-72-eGFP | 2 months | 2 w.p.i. | 1000nl | DG |
| CBR04672 | Sprague Dawley | rAAV 2/1 vHC-20-72-eGFP | 2 months | 2 w.p.i. | 1000nl | DG |
| CBR04148 | Sprague Dawley | rAAV 2/1 hSyn-Arc-N-Lobe | 2 months | 2 w.p.i. | 1000nl | DG |
| CBR04149 | Sprague Dawley | rAAV 2/1 hSyn-Arc-N-Lobe | 2 months | 2 w.p.i. | 1000nl | DG |
| CBR04239 | Sprague Dawley | rAAV 2/1 hSyn-Arc-N-Lobe | 2 months | 2 w.p.i. | 1000nl | DG |
| CBR04411 | Sprague Dawley | rAAV 2/1 hSyn-Arc-N-Lobe | 3 months | 2 w.p.i. | 1000nl | DG |
| **Coordinates**   - **AP:** -3.9mm; **ML:** +/-2.2mm; **DV:** -3.3mm | | | **Viral titer**  rAAV 2/1 vHC-20-72-eGFP: 5.6x10^12^ viral genomic particles/ml  rAAV hSyn-Arc-N-Lobe: 1.7x10^11^ viral genomic particles/ml | | | |

| **Table S1.D: Stereotaxic injections in pups mice (N=13)** | | | | | | | |
| --- | --- | --- | --- | --- | --- | --- | --- |
| **Animal ID** | **Breed** | **Virus** | **Injected at** | **Perfused at** | **Left Hemisphere** | **Right Hemisfere** | **Target Brain region** |
| 65195 | Rbp4-Cre+/TdTom + | rAAV 2/1 vHC-20-72-eGFP | P0 | P10 | 25nl | 50nl | HC formation |
| 65196 | Rbp4-Cre-/TdTom + | rAAV 2/1 vHC-20-72-eGFP | P0 | P10 | 25nl | 50nl | HC formation |
| 65197 | Rbp4-Cre+/TdTom + | rAAV 2/1 vHC-20-72-eGFP | P0 | P10 | 25nl | 50nl | HC formation |
| 65198 | Rbp4-Cre+/TdTom + | rAAV 2/1 vHC-20-72-eGFP | P0 | P10 | 25nl | 50nl | HC formation |
| 65199 | Rbp4-Cre-/TdTom + | rAAV 2/1 vHC-20-72-eGFP | P0 | P10 | 25nl | 50nl | HC formation |
| 65200 | Rbp4-Cre+/TdTom + | rAAV 2/1 vHC-20-72-eGFP | P0 | P10 | 25nl | 50nl | HC formation |
| 65201 | Rbp4-Cre+/TdTom + | rAAV 2/1 vHC-20-72-eGFP | P0 | P10 | 25nl | 50nl | HC formation |
| 65081 | Rbp4-Cre+/TdTom + | rAAV 2/1 vHC-20-72-eGFP | P0 | P30 | 25nl | 50nl | HC formation |
| 65082 | Rbp4-Cre+/TdTom + | rAAV 2/1 vHC-20-72-eGFP | P0 | P30 | 25nl | 50nl | HC formation |
| 65083 | Rbp4-Cre+/TdTom + | rAAV 2/1 vHC-20-72-eGFP | P0 | P30 | 25nl | 50nl | HC formation |
| 65084 | Rbp4-Cre+/TdTom + | rAAV 2/1 vHC-20-72-eGFP | P0 | P30 | 25nl | 50nl | HC formation |
| 65085 | Rbp4-Cre+/TdTom + | rAAV 2/1 vHC-20-72-eGFP | P0 | P30 | 25nl | 50nl | HC formation |
| 65086 | Rbp4-Cre+/TdTom + | rAAV 2/1 vHC-20-72-eGFP | P0 | P30 | 25nl | 50nl | HC formation |
| **Coordinates**  **AP**: +0.8mm; **ML:** +/- 1.2mm; **DV:** -1.22mm | | | **Viral titer**  rAAV 2/1 vHC-20-72-eGFP: 5.6x10^12^ viral genomic particles/ml | | | | |
