# Supplementary material for: Generation of an enhancer-driven gene expression viral tool specific to dentate granule cell-types through direct hippocampal injection
Source: Front Neurosci. 2024 Mar 14;18:1274174. doi: 10.3389/fnins.2024.1274174 (PMC10976853; doi:10.3389/fnins.2024.1274174)
Supplement: Supplementary file 5 [file Table_2.DOCX]

**Table S2**

| Enhancer | Tissue | Genomic coordinates | Gene in proximity of the putative enhancer sequence |
| --- | --- | --- | --- |
| vHC-20-72 | Ventral Hippocampus | mm10:chr4:15550619-15551270 | Calb |
| vHC-20-76 | Ventral Hippocampus | mm10:chrX: 166734052-166735369 | Tmsb4x |
| vHC-20-77 | Ventral Hippocampus | mm10:chr7: 118386338-118387117 | Syt17 |
| vHC-20-80 | Ventral Hippocampus | mm10:chr6: 135552239-135553116 | Grin2b |
| vHC-20-89 | Ventral Hippocampus | mm10:chr16: 41820081-41820930 | Gap43 |
| vHC-20-110 | Ventral Hippocampus | mm10: chr16: 22632633-22633429 | Dgkg |
| vHC-20-111 | Ventral Hippocampus | mm10: chr1: 164641435-164642836 | Atp1b1 |
| vHC-20-112 | Ventral Hippocampus | mm10:chr19: 57847967-57848595 | Gfra1 |
| vHC-20-143 | Ventral Hippocampus | mm10: chr2: 76282751-76285617 | Pde11a |
| vHC-20-154 | Ventral Hippocampus | mm10:chr8: 102771084-102772729 | Cdh11 |
| vHC-20-174 | Ventral Hippocampus | mm10: chr18: 65848485-65849229 | Grp |
| vHC-20-220 | Ventral Hippocampus | mm10: chr12: 38397245-38400988 | Dgkb |
| vHC-20-225 | Ventral Hippocampus | mm10: chr13: 73850251-73852459 | Nkd2 |
| vHC-20-240 | Ventral Hippocampus | mm10: chr3: 82651467-82653589 | Npy2r |
| vHC-20-243 | Ventral Hippocampus | mm10: chr12: 28640787-28642430 | Rps7 |
| vHC-20-246 | Ventral Hippocampus | mm10:chr19: 57847967-57848595 | Dio3 |
| vHC-20-257 | Ventral Hippocampus | mm10: chr1: 130084184-130087906 | Thsd7b |
| vHC-20-261 | Ventral Hippocampus | mm10: chr4: 70484283-70484992 | Megf9 |
| vHC-20-267 | Ventral Hippocampus | mm10:chr3: 152675746-152677973 | Ak5 |
| vHC-20-269 | Ventral Hippocampus | mm10:chr16: 43486927-43490785 | Zbtb20 |
| vHC-20-271 | Ventral Hippocampus | mm10: chr5: 131219903-131220534 | Wbscr17 |
